# Applying Artificial Intelligence to Childhood Obesity: T2DM and MASLD Risk Predictive Models

**DOI:** 10.3390/diagnostics16162533

**Published:** 2026-08-11

**Authors:** Marianna Amitrano, Gianluca Mondillo, Mario Emiliano, Umberto Paolo Santoro

**Affiliations:** 1 Unit of Pediatrics and Neonatology, Hospital “San Giovanni di Dio”, 80027 Frattamaggiore, Italy; 2 Department of Woman, Child and of General and Specialized Surgery, Università Degli Studi Della Campania “Luigi Vanvitelli”, 80138 Naples, Italy; gianluca.mondillo@gmail.com; 3 Department of Public Health, University of Naples “Federico II”, 80131 Naples, Italy

**Keywords:** artificial intelligence, childhood obesity, type 2 diabetes, MASLD

## Abstract

Pediatric obesity is a complex, multifactorial pandemic with serious early-onset comorbidities, including prediabetes, type 2 diabetes, metabolic dysfunction-associated steatotic liver disease (MASLD), and cardiovascular disorders. While lifestyle modifications and the Mediterranean diet remain primary interventions, artificial intelligence (AI) is emerging as a critical tool for early diagnosis and personalized management. This review evaluates the current role of AI in predicting and treating childhood obesity and its complications. A literature search was conducted on PubMed and Google Scholar for English-language articles published from 2015 onward. Search terms included combinations of keywords related to “obesity”, “pediatric”, “comorbidities” (e.g., MASLD, diabetes), and “artificial intelligence” (e.g., machine learning, deep learning, multi-omics). Eligible study types ranged from original articles to systematic reviews and clinical guidelines. By integrating multi-omic data (genome, epigenome, transcriptome, metabolome, microbiota) with socio-psychological metrics, AI can predict obesity risk and early complications. Machine learning (ML) and deep learning have successfully identified specific metabolites, gut flora alterations, neurological pathways, and metabolic SNPs linked to obesity susceptibility. Furthermore, ML-driven prognostic models enable risk assessment for MASLD or diabetes progression, while specialized software supports remote lifestyle monitoring and tailored dietary interventions. AI has the potential to revolutionize pediatric obesity management through precision medicine. However, challenges regarding data privacy, digital literacy, and equitable access persist. Because current evidence relies heavily on limited and heterogeneous pediatric datasets, large-scale, well-characterized, and externally validated cohorts are essential to establish the clinical applicability of AI models before routine implementation.

## 1. Introduction

Childhood obesity is a major global public health challenge because of its increasing prevalence and its long-term health consequences. In pediatric-age individuals, obesity is defined using age-specific WHO growth charts based on weight-for-length or body mass index (BMI) percentiles [1]. The vast majority of cases are classified as primary obesity, a polygenic and multifactorial condition resulting from the interaction of genetic susceptibility, epigenetic programming, neuroendocrine regulation, and environmental, behavioral, and psychosocial determinants [1,2,3,4,5,6,7,8,9]. Secondary forms, including monogenic and syndromic obesity, are rare but should be suspected in children presenting with early onset, rapid weight gain, short stature, developmental delay, dysmorphic features, or other syndromic manifestations [1]. Genome-wide association studies have identified susceptibility variants in genes involved in appetite regulation and energy homeostasis (including *FTO*, *MC4R*, *POMC*, *PCSK1*, and *BDNF*), while epigenetic mechanisms during the first 1000 days of life and neuroendocrine alterations, such as leptin resistance and impaired gut hormone signaling, further contribute to disease development [3,4,5,6,7,8].

The greatest clinical burden of childhood obesity derives from its metabolic complications, which develop early in life and frequently persist into adulthood [1]. Excess adiposity induces chronic low-grade inflammation characterized by macrophage infiltration of visceral adipose tissue, altered adipokine secretion, oxidative stress, and mitochondrial dysfunction, ultimately promoting systemic insulin resistance [10,11]. Elevated circulating free fatty acids and pro-inflammatory cytokines, together with reduced adiponectin levels, impair glucose uptake and increase pancreatic β-cell demand, thereby creating a predisposition to type 2 diabetes mellitus (T2DM) [10,11]. In genetically susceptible individuals, several loci associated with pediatric T2DM have been identified, although environmental factors, particularly hypercaloric diets and physical inactivity, remain the principal triggers of disease onset [12]. Compared with adult-onset disease, pediatric T2DM follows a more aggressive clinical course, characterized by greater insulin resistance, accelerated β-cell failure, poorer glycemic control, and earlier development of microvascular and macrovascular complications [11,12].

Obesity-related metabolic dysfunction also encompasses hypertension, dyslipidemia, systemic inflammation, oxidative stress, and increased cardiovascular risk, which collectively constitute the pathophysiological substrate of metabolic syndrome (MS) [10,11,12,13,14]. Although a universally accepted pediatric definition of MS is still lacking, the clustering of these abnormalities substantially increases the risk of future cardiovascular disease and premature mortality [13,14,15].

Metabolic dysfunction-associated steatotic liver disease (MASLD), formerly non-alcoholic fatty liver disease, represents the hepatic manifestation of obesity-related metabolic dysfunction and is currently the most common chronic liver disease in children with obesity [16]. Approximately one-third of obese children are affected, with disease severity ranging from simple steatosis to metabolic-associated steatohepatitis (MASH), fibrosis, and cirrhosis. Obesity, insulin resistance, dyslipidemia, hypertension, and genetic variants, particularly the *PNPLA3* I148M polymorphism, are the principal risk factors for disease progression [16]. Although advanced fibrosis is uncommon during childhood, pediatric MASLD is associated with an increased lifetime risk of progressive liver disease, cardiovascular disease, T2DM, and premature mortality, underscoring the importance of early identification and intervention [16].

Overall, the metabolic consequences of childhood obesity begin long before adulthood and represent the principal determinants of long-term morbidity. Early recognition of metabolic dysfunction, together with timely multidisciplinary interventions targeting lifestyle, behavioral, and, when appropriate, pharmacological treatment, is therefore essential to improve both metabolic health and future cardiovascular outcomes [1,10,11,12,13,14,15,16].

### Artificial Intelligence Applied to Healthcare: Available Data Sources

Artificial intelligence (AI) encompasses a broad range of computational methods that perform tasks typically requiring human intelligence, ranging from symbolic and rule-based systems to machine learning (ML) approaches capable of learning patterns from data and generating predictions or decisions that support human judgment. In healthcare, AI has emerged as a powerful tool for developing predictive models that estimate clinically relevant outcomes by integrating large volumes of heterogeneous patient data. These models are increasingly used to support diagnosis, prognosis, risk stratification, treatment selection, and personalized patient management, thereby contributing to the implementation of precision medicine [17,18].

The performance of AI-based predictive models relies on the availability of large, high-quality datasets representative of the target population. During the training process, algorithms learn the complex relationships between patient characteristics and predefined clinical outcomes, enabling subsequent prediction in previously unseen patients. Healthcare datasets typically include demographic information, medical history, laboratory tests, physiological measurements, imaging data, histopathological findings, genomic and epigenomic profiles, electronic health records (EHRs), and data generated by wearable devices or continuous monitoring systems. The integration of these multimodal data sources represents one of the major challenges in predictive modeling because they differ substantially in structure, dimensionality, and format. Advances in AI have enabled increasingly effective methods for harmonizing these heterogeneous data and extracting clinically meaningful patterns that may not be apparent using conventional statistical approaches [18].

ML, a major branch of AI, provides the methodological framework for constructing predictive models by learning the relationship between input variables and clinical outcomes directly from data. Depending on the availability of labeled outcomes, ML algorithms can be categorized as supervised, unsupervised, semi-supervised, or reinforcement learning. In clinical prediction, supervised learning is the most widely adopted approach because it uses labeled datasets to estimate predefined outcomes, such as disease diagnosis, treatment response, disease progression, recurrence, survival, or the occurrence of adverse clinical events [17,18]. Common supervised algorithms include logistic regression (LR), decision trees (DTs), support vector machines (SVMs), naïve Bayes classifiers, artificial neural networks (ANNs), and ensemble methods such as random forests (RFs) and gradient boosting (GB). These algorithms differ in their mathematical assumptions and complexity but share the objective of maximizing predictive accuracy while maintaining generalizability to independent patient cohorts.

Unsupervised learning, in contrast, identifies hidden structures within unlabeled datasets without predefined outcome variables. Although not directly predictive, it plays an important role in predictive modeling by identifying patient subgroups, reducing data dimensionality, and discovering previously unrecognized disease phenotypes. Techniques such as clustering and principal component analysis (PCA) are frequently employed during data preprocessing to improve the performance and interpretability of subsequent supervised predictive models [18]. Reinforcement learning represents another learning paradigm in which an agent optimizes sequential decision-making through interactions with its environment, making it particularly attractive for applications requiring dynamic treatment optimization and adaptive clinical decision support [17].

Deep learning (DL), a specialized subfield of ML based on multilayer artificial neural networks, has substantially expanded the capabilities of predictive modeling by learning hierarchical feature representations directly from raw data without requiring manual feature engineering. This characteristic makes DL particularly effective for analyzing complex, high-dimensional, and heterogeneous biomedical datasets. Compared with traditional ML algorithms, DL models can learn features directly from raw, high-dimensional data and have been increasingly applied to integrate multiple data modalities—including radiological and histopathological images, genomic information, laboratory measurements, physiological signals, and clinical variables—into unified predictive frameworks capable of capturing highly nonlinear relationships, although robust multimodal integration (e.g., modality alignment and missing modalities) remains an open methodological challenge [18,19].

Among DL architectures, convolutional neural networks (CNNs) have long been the reference standard for image-based predictive modeling, although vision transformers (ViTs) and hybrid architectures have more recently matched or surpassed them in several medical imaging tasks. CNNs automatically extract relevant imaging features and have demonstrated excellent performance in disease detection, lesion segmentation, tumor classification, prediction of treatment response, and prognosis across several medical specialties, including radiology, pathology, dermatology, and ophthalmology [18,19]. Their widespread adoption has been facilitated by transfer learning, whereby models pretrained on large image repositories are fine-tuned using relatively small medical datasets, improving predictive performance while reducing the amount of annotated clinical data required for training.

In addition to imaging, predictive models increasingly exploit information contained in unstructured clinical documents through natural language processing (NLP). NLP encompasses rule-based, ML, and DL approaches designed to convert narrative clinical information—including electronic health records, pathology reports, radiology reports, and clinical notes—into structured variables suitable for predictive analysis [17,18]. Modern transformer-based language models and other deep learning architectures have substantially improved the extraction of clinically relevant information from free-text documents, allowing predictive models to incorporate textual information alongside structured clinical variables.

The integration of structured clinical data, medical imaging, genomic information, physiological signals, and narrative clinical text into multimodal AI models represents one of the most promising developments in predictive medicine. Such models can simultaneously exploit complementary sources of information to improve diagnostic accuracy, estimate individualized disease risk, predict therapeutic response, identify patients at high risk of complications, and support personalized treatment strategies. In genomics, for example, DL-based predictive models have shown considerable promise in integrating genomic variants with phenotypic, radiological, histopathological, and longitudinal clinical data to predict disease susceptibility, mutation pathogenicity, biomarker expression, and clinical outcomes. As increasingly large multimodal datasets become available, AI-driven predictive modeling is expected to play an expanding role in precision medicine by enabling more accurate, individualized, and clinically actionable predictions [18,19].

**Figure 1:**
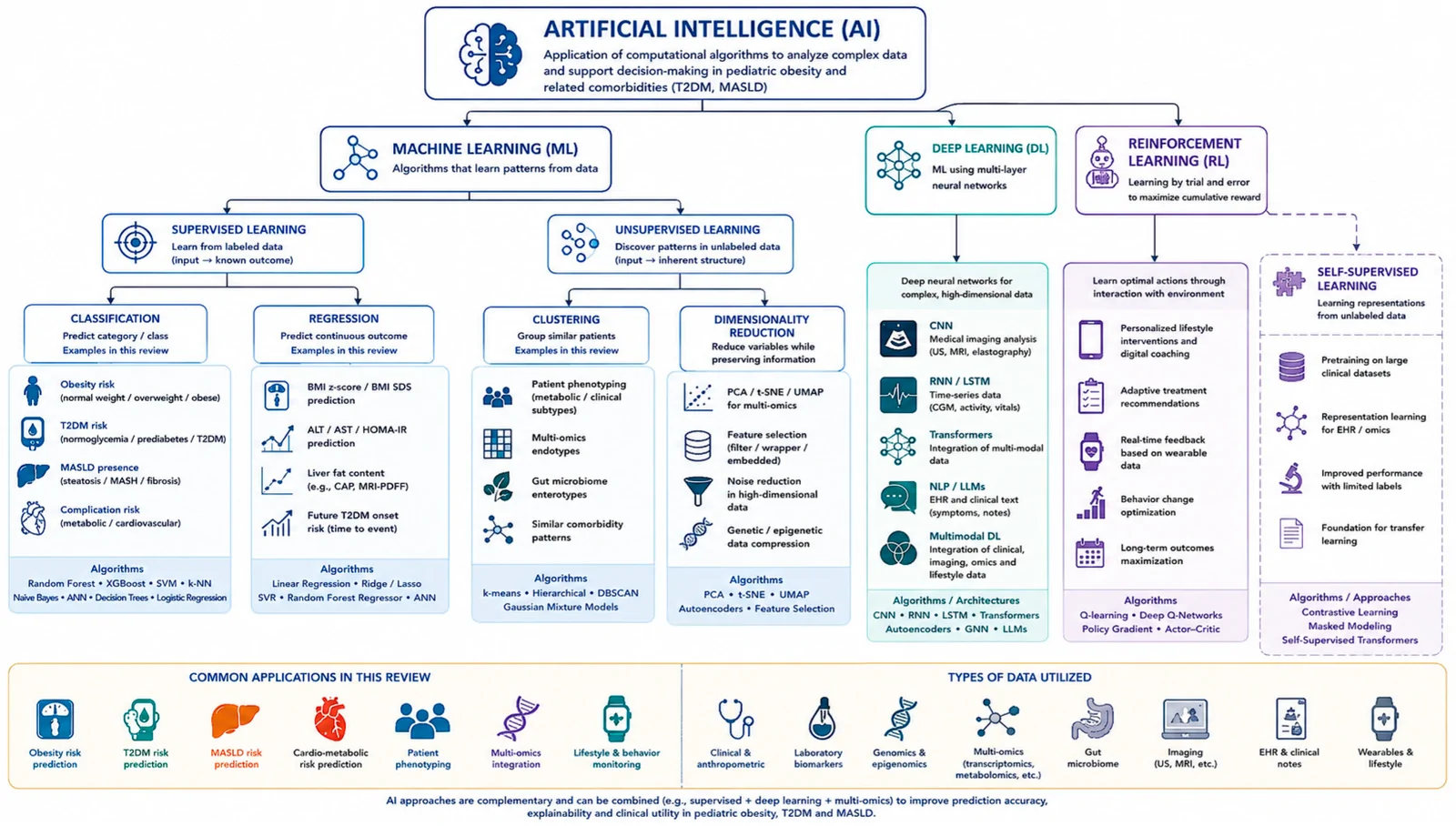
AI algorithms and their application to childhood obesity.

Figure 1 highlights the applications of AI to childhood obesity.

## 2. Methods

This narrative review was conducted through a literature search in the databases PubMed and Google Scholar. The study was conducted from December 2025 to March 2026. The search strategy combined the following keywords using the Boolean operators AND and OR: (“obesity” OR “globesity”) AND (“children” OR “pediatric”) AND (“comorbidities” OR “metabolic comorbidities” OR “MASLD” OR “diabetes”) AND (“artificial intelligence” OR “machine learning” OR “deep learning” OR “multiomics”). Additional manual searches of the reference lists of relevant articles were also performed to identify further eligible publications. The inclusion criteria were articles published in English from 2010 onward and full-text original articles, narrative reviews, systematic reviews, scoping reviews, meta-analyses, consensus statements, and clinical guidelines that are relevant to the aims of the review. Model-development studies with internal validation were included. Studies on cohorts of obese adults and cohorts of obese adults with metabolic comorbidities, such as T2DM and MASLD, and related complications were included. Studies not directly related to the topic or not meeting the inclusion criteria were excluded. Duplicate studies were excluded. Articles focusing on non-metabolic complications of childhood obesity were excluded, as were articles whose study populations consisted of older adults with other obesity comorbidities than metabolic types; studies on lean adults with MASLD were also excluded. Article selection was based on title and abstract screening, followed by full-text assessment when appropriate. Screening and data extraction were performed independently by two different authors. A total of 96 papers were selected in the screening phase. Data about obesity prediction risk, metabolic complication prediction risk, and AI-based models used to implement clinical treatment and monitoring of obesity and its metabolic complications were extracted; when none of these was available the paper was excluded (9 papers). All the authors agreed to the inclusion of the selected papers and to the exclusion of the others.

## 3. Results

Several scientific works in the literature have demonstrated the usefulness of AI in various fields of healthcare and in recent years AI models have been applied to different aspects of childhood obesity. In particular, AI techniques have been used for the assessment of nutritional status, early diagnosis of complications, the therapeutic response to drugs and bariatric surgery, and the formulation of personalized lifestyle-modification treatments [20]. This results in a significant contribution of AI to precision medicine, which will be able to provide personalized treatments to patients based on their risk category and will be able to direct pharmacological treatment to those patients who are most likely to benefit from it. The goal of precision medicine in childhood obesity is to identify a targeted treatment based on a precise diagnosis based on the individual patient’s specific characteristics: genetic and epigenetic factors, environmental factors such as diet, environmental pollutants, a sedentary lifestyle, smoking, insomnia (which constitute the exposome, i.e., the set of factors to which the individual is exposed), and metabolic and meta-inflammatory status. This process of integrating the patient’s global characteristics is called “deep phenotyping” and allows for the creation of a risk profile for each patient and the individualization of treatment strategies [10].

**Figure 2:**
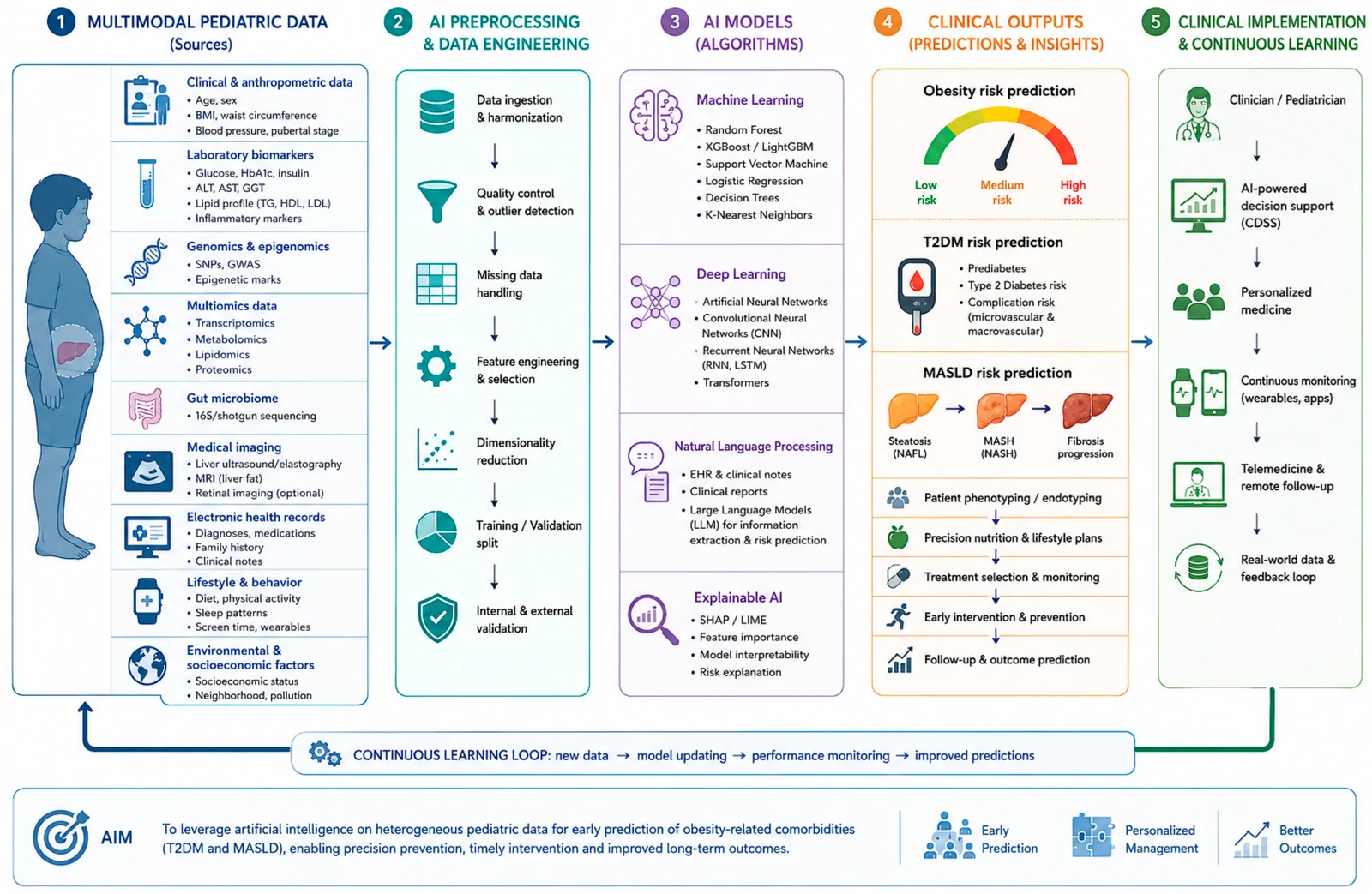
AI application in healthcare: integration of multimodal data (e.g., genetic, epigenetic, clinical and biochemical biomarkers, microbiota, radiological data, EHR, behavioral and socioeconomic data), generating algorithms that allow risk prediction for a certain outcome, in this case obesity-related metabolic complications. Risk prediction models can create specific dietary and monitoring plans, allowing precision treatment and preventive strategies.

Figure 2 synthesizes AI applications in healthcare and specifically to childhood obesity and its metabolic complications.

AI-based models integrate heterogeneous data, including EHRs, growth trajectories, lifestyle factors, dietary habits, physical activity, and social and environmental determinants, improving obesity risk stratification and prevention strategies [20]. Computer vision applied to satellite imagery can quantify obesogenic environmental features, such as green spaces, recreational facilities, bike paths, and the density of fast-food outlets, enabling the generation of high-resolution maps of obesity risk areas to support targeted public health interventions [21].

ML and DL algorithms, including recurrent neural networks (RNNs), can model the complex interactions underlying childhood obesity. AI has been used to integrate neuroimaging and metabolomic data, identifying structural brain alterations in reward- and default-mode networks and microbiome-derived metabolites associated with obesity, achieving high diagnostic accuracy in distinguishing obese from overweight and severely obese individuals [22]. These findings are supported by preclinical evidence demonstrating altered brain activity and microbiome composition in obesity, highlighting the role of the gut–brain axis and amino acid-derived metabolites such as agmatine [23,24].

AI also supports the identification of obesity-related psychopathological comorbidities. For example, a naïve Bayes predictive model based on the Yale Food Addiction Scale can identify patients with food addiction who may benefit from targeted psychological interventions, potentially improving treatment adherence and outcomes [22].

Numerous ML and DL models have been developed to predict obesity onset, disease progression, and obesity-related complications by integrating clinical, environmental, behavioral, genomic, and epigenomic data [19,25,26,27,28,29]. Among the most accurate approaches are artificial neural networks (ANNs), SVMs, RF, DTs, and k-nearest neighbors (KNNs), which have demonstrated good performance in identifying high-risk individuals and relevant biomarkers [19,27]. Multi-omics approaches combining genomic variants, DNA methylation, dietary factors, and lifestyle characteristics further support precision prevention strategies from a nutrigenomics perspective [25]. AI-based clinical decision-support systems have also identified early-life and socioeconomic risk factors for pediatric obesity, including rapid weight gain, family characteristics, and obesogenic environments [28,30]. In addition, ML has been applied to personalized meal planning, prediction of metabolic complications, and drug response through protein–protein interaction modeling [31,32,33].

AI is increasingly being integrated into obesity management through digital health technologies, including mobile applications, wearable devices, digital coaches, large language models (LLMs), digital therapeutics (DTx), exergames, and Internet of Things (IoT)-based platforms [33,34,35,36,37,38]. These tools provide personalized recommendations for diet, physical activity, sleep, and behavioral change, while enabling continuous monitoring and remote clinical supervision through reinforcement learning algorithms and wearable sensors. Platforms such as ETIOBE integrate patient-generated data with clinical information to support individualized treatment and long-term follow-up [38]. Although these technologies show promising effects on adherence, lifestyle modification, and weight management, their effectiveness remains dependent on user engagement, digital literacy, and the reliability of AI-generated recommendations [33,34,35,36,37,38]. Consistent with the 2023 American Academy of Pediatrics guidelines, AI-based tools should complement comprehensive, multidisciplinary lifestyle interventions rather than replace them [39].

### 3.1. Predictive Models for the Diagnosis and Management of T2DM

Various AI techniques have been applied for the diagnosis and risk stratification of complications in obese patients. In particular, this section and the next one focus on the use of AI for the early diagnosis and the formulation of predictive models for the risk of T2DM and MASLD, given the high prevalence in obese children and adolescents, which justifies the extensive study by several research groups in the last decade.

Validated applications of ML and DL in the diagnosis and management of T2DM are diverse: through DTs, RF, or neural networks, it is possible to predict the risk of diabetes in obese patients by analyzing lifestyle, clinical and psychological factors, and physical and social habits; and they can be used to build predictive prognosis models and personalize treatment in diabetic patients and to predict long-term complications; for example, through DL (CNN) it is possible to perform automated retinal screening to identify early signs of diabetic retinopathy. Furthermore, it is possible to phenotype the patient through genomic and epigenetic studies and achieve autonomous patient management through monitoring devices that use AI software, sensors and telemedicine [40].

However, as shown in Table 1, research by Yang et al. produced an ML model to predict the onset of T2DM in obese children: 292 children with obesity and T2DM were enrolled and their characteristics were studied. Eight ML models were compared for their ability to identify clinical and biochemical characteristics for the creation of predictive risk models. The SVM was the best model for predicting the onset of T2DM in obese children and identified eight characteristics on which the predictive model was based: BMI, creatinine, prealbumin, thyrotropin, total thyroxine, free thyroxine, glycosylated hemoglobin, and blood glucose 180 min after an oral glucose load [41]. In contrast, many more studies have been conducted on cohorts of obese adult patients. The study by Zou et al., conducted on a large cohort of patients, developed an RF-based predictive model that forecasts the risk of developing T2DM based on the patients’ clinical and biochemical characteristics [40]. Using an ML algorithm, the study by Sun et al. analyzes dietary patterns and identifies, among various diets, the one associated with obesity and the highest risk of T2DM, based on sugary and processed foods [42]. The review by Nomura et al. reports articles demonstrating that using models based on GB, RF and LR it is possible to predict the onset of T2DM up to 5 years before the onset [43]. Techniques such as ANN applied to a database of biochemical data have been found to be effective in identifying patients with prediabetes and T2DM [44]. Models using DT and RF have shown good specificity and accuracy in associating risk factors such as high total cholesterol, LDL cholesterol, and triglycerides with the onset of T2DM, as well as less-considered risk factors such as having fewer than 6 h of sleep a night, a high sodium intake, psychological stress, exposure to environmental pollutants, and living in urbanized areas [45]. Furthermore, through ML it was possible to study and identify biomarkers of beta-cell dysregulation in patients with T2DM, such as the depletion of mature insulin-producing cells, the expansion of immature cells and of cells produced in response to endoplasmic reticulum stress, and the modification of acinar cells towards an inflammatory pattern and of ductal cells towards a secretory pattern [46].

ML is effective in producing predictive models of risk of developing complications in obese patients with T2DM [47]. Already in 2018, Ahlqvist and colleagues had applied k-means and hierarchical clustering to reclassify adults with new-onset diabetes into subgroups based on clinical and biochemical characteristics, comparing the groups using LR and Cox regression methods to compare the risk of complications across groups. Among the subgroups, individuals with insulinopenia showed a higher risk of diabetic retinopathy, while individuals with severe insulin resistance had a higher risk of diabetic nephropathy [48]. Another k-means-based model was used in a cohort of 19,084 individuals with T2DM, classified into four groups based on clinical variables (age at diagnosis, BMI, glycosylated hemoglobin, HDL cholesterol, C-peptide, waist circumference), identifying a group with early-onset T2DM with poor glycemic control and a high risk of nephropathy and retinopathy [49].

ML has been widely applied in the field of precision nutrition to tailor a personalized diet to the individual, aimed at preventing or managing diet-related diseases [50,51,52]. An example is provided by the study by Zeevi et al., who developed a personalized diet to predict glycemic response in 800 healthy and prediabetic adults, considering biochemical and anthropometric data, diet, physical activity, and gut microbiota data in an integrated approach. The researchers adopted a GB regression (ML) algorithm that accurately predicted postprandial glycemic responses to meals: a randomized, blinded, controlled intervention based on an algorithm-predicted diet led to significantly lower postprandial glycemic values and changes in gut microbiota composition [51]. Similar results were obtained in two other similar studies on a population of American adults [53,54]. In the therapeutic management of T2DM, a platform (AdvisorPro manufactured by DreaMed Diabetes, Ltd., https://clinicaltrials.gov/study/NCT04043260 (accessed on 5 August 2026)) has been created that integrates data from continuous monitoring sensors with self-monitoring of blood glucose levels to suggest insulin dose adjustments, with results no lower than those proposed by the clinician. Another software program (Guardian manufactured by Medtronic, https://carelink.medtronic.eu/ (accessed on 5 August 2026)) allows for the prediction of hypoglycemia in diabetic patients up to 30 min in advance, allowing for better glycemic control and fewer adverse events [43].

**Table 1:** Machine learning and deep learning models for type 2 diabetes mellitus and its complications: expanded study characteristics.

Article	Study Design: Prospective or Retrospective	Population: Exact Age Range and Characteristics	Model-Development Sample; Validation Sample; Internal and External Validation	Outcome Definition and Reference Standard	Predictor Variables	Algorithm	Discrimination Metrics	Calibration	Interpretability/Explainability	Clinical Availability/Deployment Status	Pediatric Applicability
Yang et al., 2025 [41]	Design: Single-center observational study; participants recruited July 2023–February 2024 with at least 1 year of follow-up, data abstracted from clinical records. NOTE: The Limitations section of the paper describes the study as retrospective, which is inconsistent with the enrolment-plus-follow-up description in the Methods section. Timing: Retrospective per the authors’ own Limitations statement; data abstracted from case records.	Children with obesity aged <18 years attending endocrinology outpatient clinics/wards, Children’s Hospital of Soochow University, Suzhou, China. *n* = 292 analyzed (300 enrolled, 8 excluded for missing data). Mean age 11.96 (SD 2.29) y; 162/292 (55.5%) male. Obesity defined by weight-for-height >20% above the reference (Zhufutang Practical Pediatrics, 8th ed), NOT by BMI z-score/percentile. No T2DM 11.79 (2.26) y vs. T2DM 12.80 (2.25) y, *p* = 0.005.	Development: *n* = 292; random split 75% training/25% internal validation; 10-fold cross-validation within the training set. 49 outcome events (16.8%) → approx. 4–5 events per candidate predictor (8 predictors). No sample size calculation reported. Validation: Internal only (random 25% hold-out set + 10-fold CV). NO external validation. Authors state multi-center external validation is planned. Internal/external: Internal: 75/25 random split plus 10-fold CV. External: None.	T2DM diagnosed by ADA/ISPAD criteria (symptoms plus FPG ≥ 7.0 mmol/L, or 2 h post-load glucose ≥ 11.1 mmol/L, or HbA1c ≥ 6.5%, or random glucose ≥ 11.1 mmol/L; abnormal values confirmed on retesting when asymptomatic). Diabetes type assigned in a second step using GAD, IA-2, ZnT8 and insulin autoantibodies, with genetic testing for MODY where indicated. Reference standard: Clinical/biochemical ADA-ISPAD criteria.	8 predictors selected by univariable comparison then logistic regression: BMI, creatinine, prealbumin, 180 min OGTT glucose, HbA1c, thyrotropin, total T4, free T4.	8 algorithms compared: DT, LR, SVM, MLP, AdaBoost, RF, GBDT, XGBoost. SVM selected as final model (RBF kernel, C = 10). SMOTE oversampling applied to the training set; median/mode imputation; standardization; one-hot encoding; grid search.	SVM AUC 0.904 with accuracy 0.9322, recall 0.600, F1 0.750, CV accuracy 0.983 . Highest AUC of any model was MLP 0.961.	Not assessed. No calibration plot, calibration slope/intercept, Brier score or Hosmer–Lemeshow test reported. A nomogram was constructed from the model results but its calibration is not reported.	None applied. No SHAP, LIME or equivalent, despite the Introduction describing the model as “interpretable”. Interpretation is limited to logistic regression coefficients and a nomogram.	None. Research prototype; no software, no regulatory approval, no prospective clinical use.	Fully pediatric (<18 y) and obesity-specific—the only such T2DM model in this review. Transportability limited by the single Chinese center, small sample, non-standard obesity definition and reliance on a 180 min OGTT sample that is not part of routine pediatric practice.
Zou et al., 2018 [40]	Design: Retrospective cross-sectional diagnostic classification study using routine hospital physical-examination records (Luzhou, China), plus a secondary analysis of the public Pima Indians Diabetes dataset. Timing: Retrospective; secondary use of routine examination records and a public dataset.	Luzhou dataset: Attendees of routine hospital physical examinations, China. After deletion of abnormal and missing records, there were 151,598 diabetes and 69,082 healthy records. Age, sex and BMI distribution not reported. Pima dataset: Females of Pima Indian heritage aged ≥ 21 y; 768 records reduced to 392 after deletion of missing data. Neither dataset is obesity-defined.	Development: Five balanced training sets, each 68,994 healthy plus 68,994 diabetes records (i.e., approx. 137,988 records per draw); results averaged over the 5 draws. NOTE: The current Table 1 entry “68,994 subjects” refers to one class only. Validation: Five-fold cross-validation (internal). Separate hold-out “independent test set” of 13,700 records drawn from a second physical-examination dataset at the same source. Pima dataset analyzed separately with 10-fold CV; it was not used to validate the Luzhou model. Internal/external: Internal: 5-fold CV plus a same-source hold-out test set. External: none (no independent population/setting).	Prevalent “diabetes mellitus” as labeled in the source database. The basis on which labels were assigned is NOT described. Diabetes type was not distinguished—the authors state explicitly that they cannot predict the type of diabetes. No T2DM-specific reference standard.	Luzhou: 14 routine indices-age, pulse rate, respiratory rate, left/right systolic and diastolic pressure, height, weight, physique index (BMI), fasting glucose, waistline, LDL, HDL. Pima: 8 standard attributes. Dimensionality reduction by PCA and mRMR.	J48 DT (WEKA), RFt, and a two-layer feed-forward neural network with 10 hidden neurons (MATLAB).	No AUC or C-statistic reported . Luzhou 5-fold CV, all features: RF accuracy 0.8084, sensitivity 0.8495, specificity 0.7673, MCC 0.6189 (best of the three classifiers). Same-source hold-out test, all features: RF accuracy 0.8963, sensitivity 0.9226, specificity 0.8700, MCC 0.7937. Pima 10-fold CV best accuracy 0.7852 (mRMR + RF).	Not assessed.	Partial: DT structures displayed, information-gain based splits, and mRMR feature ranking. No SHAP/LIME or other post hoc explanation method.	None. The Pima dataset was made available online; no model, software or tool was deployed.	None. Age not reported for the Luzhou dataset; the Pima dataset is restricted to women aged ≥ 21 y. No pediatric data and no obesity-specific analysis.
Sun et al., 2025 [42]	Design: Cross-sectional community-based study, Heze City, Shandong, China; recruitment 2018–2019 by multistage stratified random sampling. Timing: Cross-sectional; data collected 2018–2019, analyzed retrospectively. No temporal separation between predictors and outcome.	982 men aged ≥ 60 y (mean approx. 74.4, SD 8.6 y). Male sex only. 453 (46.1%) rural, 529 (53.9%) urban. Mean BMI 26.7–26.9 kg/m^2^; general obesity present in 52% and central obesity in 46–51%, but the cohort is NOT obesity-selected. Exclusions: Type 1 diabetes, severe cardiovascular/renal/hepatic disease, cognitive impairment, metabolically active medication.	Development: 982 participants; no separate development/validation split. XGBoost trained on the full dataset with 5-fold cross-validation and grid-search hyperparameter tuning. Validation: Internal only (5-fold CV plus sensitivity analyses stratified by BMI and energy-intake quartile). The authors state explicitly that no independent external validation dataset was available. Internal/external: Internal: 5-fold CV. External: None.	T2DM defined by ADA criteria: FPG ≥ 7.0 mmol/L, HbA1c ≥ 6.5%, self-reported physician diagnosis, or current antidiabetic medication. Prevalence reported as 48.37% “newly diagnosed”. CAUTION: The paper’s own Table 1 shows no difference in FPG (135.4 vs. 135.0 mg/dL, *p* = 0.39) or HbA1c (6.96 vs. 7.02%, *p* = 0.16) between the T2DM and non-T2DM groups, and approximately 50% of both groups were taking antidiabetic medication—internally inconsistent with the stated outcome definition and with the “newly diagnosed” label.	Data-driven dietary patterns (81-item validated semi-quantitative FFQ collapsed into 21 food groups, then UMAP + k-means clusters: high-fiber nutrient-dense, staple-protein, seafood-eggs, sugary and processed foods), total energy intake, physical activity (IPAQ, MET-h/week), demographics, anthropometry, blood pressure, and biochemistry.	UMAP (dimensionality reduction) + k-means (dietary pattern clustering); XGBoost classifier for T2DM; SHAP for model interpretation; multivariable logistic regression for associations.	XGBoost, 5-fold CV: Mean ROC AUC 0.83, accuracy 84%). No discrimination metric is currently reported for this study	Not assessed.	SHAP applied (summary, waterfall and force plots) identifying dietary patterns, total energy intake and physical activity as the leading contributors.	None.	None. Men aged ≥ 60 y only; the authors note that generalizability to women and other age groups is unknown. Least-applicable study in Table 1 to a pediatric obesity review.
Nomura et al., 2021 [43]	Design: Retrospective analysis of annual specific health checkup records, Kanazawa City, Japan, 2008–2018. Timing: Retrospective.	509,153 annual health checkup records from 139,225 individuals; 65,505 individuals without diabetes at baseline formed the analysis set. Age range, sex distribution, BMI and obesity status not reported in the paper.	Development: 65,505 individuals split 6:2:2 into training (36,303), tuning (13,101) and testing (13,101). 4696 incident diabetes cases (7.2%). Validation: Internal only (hold-out test set, with 1000-iteration bootstrap for confidence intervals). No external validation. Internal/external: Internal: 6:2:2 split with bootstrap CIs. External: None.	New onset of diabetes mellitus during annual health checkups. The precise diagnostic criteria and whether T2DM was distinguished are not reported in the paper.	Physical examination measurements, blood and urine tests, and participant questionnaires from the annual checkups. The specific variables entered into the model are not reported in the paper.	Gradient-boosting DTs.	Test set: AUC 0.71 (95% CI 0.69–0.72); precision 75.3% (71.6–78.8); recall/sensitivity 42.2% (39.3–45.2); F1 54.1% (51.2–56.7); accuracy 94.9% (94.5–95.2). NOTE: Accuracy is inflated by the 7.2% event rate and should not be read as a measure of usefulness.	Not reported.	Not reported.	Not reported.	None—annual specific health checkups in Japan target adults (≥40 y for the standard program). No pediatric data.
Cardozo et al., 2022 [44]	Design: Retrospective cross-sectional diagnostic/screening study using a routine clinical laboratory database (Santa Luzia Medical Laboratory, Florianopolis, Brazil). Timing: Retrospective; secondary use of an existing laboratory database.	62,496 patients undergoing routine laboratory testing. Age 19–99 y; mean 56.7 (SD 16.2) y; 43.4% male, 56.6% female. HbA1c categories: 47.60% healthy, 37.95% prediabetes, 14.45% diabetes. Not obesity-selected; BMI was not available (laboratory variables only).	Development: 80% of 62,496 (approx. 49,997) for training, of which 30% was reserved for hyperparameter tuning by Bayesian optimization with a Gaussian process. Validation: Internal only: 20% random hold-out test set (approx. 12,499). No external validation. Internal/external: Internal: 80:20 split with a nested tuning subset. External: None.	Glycated hemoglobin category as the reference standard: healthy <5.7%, prediabetes 5.7–6.4%, diabetes ≥ 6.5%. Single HbA1c measurement without confirmatory testing. Diabetes type NOT distinguished; the target is HbA1c-defined diabetes status, not T2DM specifically.	15 routine laboratory variables selected by factor analysis: age, creatinine, fasting plasma glucose, basophils %, MCHC, MCH, haematocrit, leucocytes, lymphocytes %, monocytes %, MPV, platelets, RDW, segmented neutrophils %, MCV. Fasting plasma glucose is itself a diabetes diagnostic test.	Five classifiers (KNN, SVM, naïve Bayes, RF, ANN) and their regression counterparts predicting HbA1c as a continuous value followed by classification; three dataset arrangements (healthy/prediabetes/diabetes; healthy vs. not healthy; no diabetes vs. diabetes).	No AUC OR C-statistic reported. Best overall result, ANN on the healthy vs. not-healthy arrangement: sensitivity 78.1%, precision 78.7%, F1 78.4%. On the no-diabetes vs. diabetes arrangement: ANN sensitivity 67.9%, specificity 97.9%, precision 84.8%; RF sensitivity 66.3%, specificity 98.1%, precision 85.7%; KNN precision 93.7% with sensitivity 42.7%. Best regression MSE 0.29 (ANN).	Not assessed.	Minimal: factor analysis for feature selection and confusion matrices. No SHAP, LIME or equivalent.	Not deployed. Proposed as a laboratory-side screening alert to trigger confirmatory HbA1c testing; the authors state explicitly that the approach is not recommended for diagnostic purposes.	None. Adults aged 19/20–99 y; no pediatric data and no obesity-related variables.
Li et al., 2025 [47]	Design: Systematic review and meta-analysis of ML-based prediction models (PRISMA-DTA, CHARMS and TRIPOD applied; protocol registered INPLASY202490038). Databases searched to 18 April 2024. NOT A PRIMARY PREDICTION MODEL STUDY. Timing: The included studies were predominantly retrospective.	26 included studies, 94 ML models. Adults with T2DM; 14 cohort, 9 cross-sectional, 3 case–control designs, predominantly retrospective and single-center. Countries: China 14, USA 4, Singapore 3, Iran 3, Italy 1, Bangladesh 1. Individual study sample sizes range from 133 to 147,664; reported mean ages of included cohorts are approximately 51–64 y. No pediatric study was included.	Development: Not applicable at the review level. Across the included studies, 81 models were assessed in internal validation sets (total internal validation sample 319,190 participants) and 13 in external validation sets. Validation: 25/26 studies performed internal validation (hold-out or k-fold); only 8/26 performed external validation—4 with genuinely independent datasets, 3 with temporal splits and 1 partially independent. Combined external validation sample 37,944 across 7 studies. Internal/external: See validation columns: internal 25/26 studies; external 8/26 studies.	Diabetic kidney disease in patients with T2DM (albuminuria and/or reduced eGFR). 18 of the included models were diagnostic and 8 prognostic; prognostic follow-up ranged from 1 year to a median of 7.8 years.	Across studies: demographics, medical history, diabetes duration, complications, lifestyle, physical examination, laboratory tests, and in some studies circulating metabolites, genetic parameters, retinal photographs, renal ultrasound and renal pathology images. Predictor selection most often by RFECV or LASSO.	Pooled across traditional regression ML, general ML and deep learning. Individual algorithms included LR, RF, DT, SVM, GBDT, XGBoost, LASSO, Bayesian networks, naïve Bayes, AdaBoost, KNN and deep learning models.	Pooled AUC 0.839 (95% CI 0.787–0.890) in internal validation sets, with I2 = 99.8% and a 95% prediction interval of 0.56 to 1.00; pooled AUC 0.830 (95% CI 0.784–0.877) in external validation sets. Subgroups: Traditional regression ML 0.797 (0.777–0.816), general ML 0.811 (0.785–0.836), deep learning 0.863 (0.825–0.900). RF was the best-performing individual algorithm, pooled AUC 0.848 (95% CI 0.785–0.911). The very wide prediction interval means performance in a new setting could be no better than chance.	Not pooled. The review reports that 14 of the 26 included studies failed to assess discrimination and calibration adequately.	Not extracted or synthesized by the review.	None of the included models had reached clinical deployment.	None. All included studies were conducted in adults with T2DM; no pediatric evidence.
Anjana et al., 2020 [49]	Design: Retrospective analysis of an electronic medical record database from a network of 50 diabetes centers across 9 Indian states, with longitudinal follow-up for complications; replication of the clustering in the nationally representative ICMR-INDIAB population survey. Unsupervised phenotyping study, NOT a prediction model study. Timing: Retrospective derivation from EMR data with prospective longitudinal follow-up for complication outcomes.	19,084 individuals with T2DM aged 10–97 y with diabetes duration <5 y at first clinic visit (mean duration 1.74, SD 1.4 y). Selection: 373,000 records with T2DM → 55,429 with complete baseline variables → 20,850 with duration <5 y → exclusion of implausible HOMA values (*n* = 1512), 5SD outliers (*n* = 188) and GAD-antibody-positive individuals (*n* = 66) → 19,084. Mean age at diagnosis by cluster 42.1–50.2 y; 58.6–73.7% male; mean BMI by cluster 24.9–32.6 kg/m^2^.	Development: 19,084 for the k-means clustering (derivation). Validation: Replication in ICMR-INDIAB, a nationally representative population-based survey across 15 Indian states: 3851 individuals with T2DM, 2204 after exclusions, clustered on 6 of the 8 variables (C-peptide unavailable). Internal/external: Internal: Bootstrap stability (Jaccard index > 0.75), silhouette width, sex-stratified re-clustering, sensitivity analyses at diabetes duration <1, <3 and <5 y. External: Replication in ICMR-INDIAB (*n* = 2204).	Not a predicted outcome but incident microvascular complications analyzed by Cox regression: retinopathy (four-field stereo color fundus photography graded by a retinal specialist using a modified ETDRS system), nephropathy (micro- or macroalbuminuria), CKD (eGFR <60 mL/min/1.73 m^2^, CKD-EPI) and diabetic kidney disease (CKD and/or albuminuria). T2DM itself defined by absence of ketosis, fasting C-peptide > 0.6 pmol/mL, absence of pancreatic calculi and response to oral agents for at least 2 y.	Eight clustering variables: age at diagnosis, BMI, waist circumference, HbA1c, serum triglycerides, HDL cholesterol, and fasting and stimulated C-peptide.	k-means clustering (k = 4, max 10,000 iterations, R 3.6.0) on scaled and centered values; Hopkins statistic for clustering tendency; silhouette width for the number of clusters; Jaccard bootstrap (2000 resamples) for cluster stability; Cox proportional hazards models for complications adjusted for age at diagnosis and sex.	Not applicable-no individual-level risk prediction model, therefore no AUC or c-statistic. Four clusters identified: SIDD 26.2%, IROD 25.9% (novel), CIRDD 12.1% (novel), MARD 35.8%. SIDD had the highest hazard for retinopathy, followed by CIRDD; CIRDD had the highest hazard for kidney disease.	Not applicable (no predicted probabilities).	Inherently interpretable: clusters are defined by eight routine clinical and biochemical variables, with published cluster means and formal stability statistics.	None. Phenotypic classification proposed for research and risk stratification, not implemented as a tool.	Minimal. The eligible age range began at 10 y, so adolescents are included, but mean age at diagnosis was 42–50 y and no pediatric or adolescent subgroup analysis was performed.
Zeevi et al., 2015 [51]	Design: Prospective cohort with continuous glucose monitoring, followed by validation in an independent cohort and a blinded randomized controlled dietary intervention. Timing: Prospective cohort with a prospective, blinded, randomized controlled evaluation of the algorithm-guided diet—the only prospectively and interventionally evaluated model in Table 1.	Main cohort: 800 individuals with week-long continuous glucose monitoring and 46,898 meals recorded. Validation cohort: An independent 100-person cohort. Exact age range, sex distribution and BMI/obesity status not available. The cohort was not restricted to people with obesity or diabetes.	Development: 800-person cohort, 46,898 meals. Validation: Independent 100-person cohort, plus a blinded randomized controlled dietary intervention based on the algorithm’s predictions. Internal/external: Internal: Cross-validation (details not available from the paper). External: Independent 100-person cohort, plus prospective randomized interventional evaluation.	Postprandial glycemic response to real-life meals, measured by continuous glucose monitoring (a continuous physiological outcome, not a disease diagnosis). Reference standard: CGM-derived incremental glucose response.	Blood parameters, dietary habits, anthropometrics, physical activity and gut microbiome features. The complete predictor list is not available.	ML (gradient boosting regression) integrating the above data types.	Not applicable in the diagnostic sense—the model predicts a continuous outcome and is reported by correlation between predicted and measured postprandial responses. Exact R/R2 values are not available from the paper.	Not available	Not available	The approach was subsequently commercialized as a personalized nutrition service, but no regulated clinical decision-support deployment is reported in this paper.	None. Adult cohort; no pediatric data.

### 3.2. Predictive Models for the Diagnosis and Management of MASLD

It is well known that there are no validated treatments for MASLD and that the gold standard for diagnosis remains a liver biopsy, an option that is not very feasible, especially in children. Thanks to ML and DL algorithms, advances have been made in screening, long-term prognosis, treatment, and monitoring of MASLD [55]. Table 2 summarizes the contribution of AI in this field.

AI has improved our understanding of the pathogenesis of MASLD in obese patients through the study of lipidomics, the transcriptome, metabolomics, and the intestinal microbiota of obese patients with MASLD. ML techniques can analyze the transcriptome and genes implicated in the pathophysiology of MASLD, identifying new genes and biomarkers associated with it, such as AXUD1, FOSB, GADD45B, and SOCS2 [56]. Metabolomics and the lipidome can be studied, integrating data from a multi-omics perspective (in this field, metabolites such as glutamic acid, isocitric acid, and C4BPA have been identified as predictors of MASLD) [57]. It is also possible to study the role of exposure to environmental pollutants, such as pesticides, in the onset and progression of MASLD, or to conduct large-scale screening using EMR to identify the true incidence of this disease. ML also allows for risk stratification, prognosis prediction, and analysis of the role of comorbidities in the overall risk of the individual patient, with a view to precision medicine. It allows for treatment support by providing a personalized diet and a prediction of drug response [58]. ML-based models, especially RF and SVM, have been validated in predicting the onset of MASLD based on clinical and/or demographic characteristics of obese patients [59,60,61,62,63,64]. In the study by Zhang et al., a predictive model based on an XGBoost algorithm was found to be effective in establishing the risk of MASLD using clinical and biochemical data as predictive factors, with particular predictive value of waist circumference [65]. In the work of Li et al., two combined indices (roundness index and triglyceride–glucose index) were identified as predictive factors of MASLD in obese patients through ML [66], while another working group realized a visceral adiposity index as a predictor of MASLD through RF and XGBoost [67]. Another model integrated factors such as age and sex with a dietary inflammation score and the presence of diagnostic features of metabolic syndrome to estimate the risk of MASLD in obese individuals: this model suggests that consumption of high glycemic index foods, low intake of flavonoids and vitamin D, low perception of one’s health status, poor psychological well-being and an increasing trend of liver enzymes are all predictors of the onset of MASLD [68]. Another ML-based model highlighted BMI and triglycerides as independent predictors of steatosis and progression to MASH [69]. Regarding the prognosis of MASLD, ML demonstrated approximately 10% better performance than the FIB4 calculation method in discriminating liver fibrosis, using clinical, anthropometric and biochemical data [70]. Through ML it is possible to identify negative prognostic factors in patients with MASLD, including the risk of developing hepatocellular carcinoma [71]. A model called CART was also created to identify subjects with MASLD at high cardiovascular risk [72].

DL-based models, such as CNN, have been shown to be useful in detecting hepatic steatosis on ultrasound images [73], in quantifying hepatic steatosis from computed tomography images [74], and in identifying microscopic features typical of MASLD from histological images [75,76,77,78]. In the study by Heinemann et al., a CNN-based model created a predictive score for steatosis, inflammation, and fibrosis from biopsy images [79]. A model using CNN has been shown to be able to recognize stages of fibrosis from elastography data. Furthermore, it is possible to integrate the data from radiomics (computed tomography, ultrasound, contrast-enhanced ultrasound, magnetic resonance imaging) with data derived from circulating DNA or biomarkers derived from metabolomics studies [80].

Regardless, to date, there have been few studies conducted on large pediatric cohorts whose data have been validated in independent cohorts. An ML model comprising SVM, Neural Net and XGBoost algorithms, tested on 132 children and externally validated in an independent cohort, showed good performance in diagnosing MASLD on ultrasound images [81]. A study by Wang et al. identified 15 circulating inflammatory proteins associated with MASLD in obese children, developing a proteomics-based risk score (ProScore) to increase the diagnostic accuracy of MASLD in overweight and obese children. In particular, a panel of six proteins (FGF21, CDCP1, CD244, OPG, FLT3L, MCP1) was found to be predictive, especially in children with low genetic risk, allowing further stratification of this group [82]. The group of Li et al. instead created a predictive nomogram in which BMI, waist circumference, fat mass and ALT were identified as risk factors positively associated with MASLD in obese children [83]. Using ML algorithms, biomarkers such as tryptophan derivatives, metabolites that induce steatosis and oxidative stress, were found to be elevated in obese patients with MASLD [84]. Some studies have analyzed the role of the microbiota in the pathogenesis of MASLD: alterations in the microbiota influence the development and severity of MASLD and MASH. AI has analyzed the microbiome from children with MASLD and MASH and many differences in species diversity were found. Using XGBoost-based models and RF, it was possible to predict the onset of MASLD in obese children and MASH in children with MASLD based on the characteristics of the microbiota [85]. A strain, *Prevotella copri*, has been identified that worsens the progression of MASLD by down-regulating lipid metabolism genes, increasing the accumulation of triglycerides and cholesterol in the liver and down-regulating the expression of occludins, increasing intestinal permeability [86]. A specific microbiota typical of MASLD has been isolated through metagenomic studies. ML identified 12 bacterial species in obese patients with MASLD, while some strains are typically less present. *Eubacterium hallii* was less abundant in patients with MASLD, both obese and lean, suggesting a possible role as a probiotic. This strain is one of the producers of short-chain fatty acids, which have an anti-inflammatory effect, improve intestinal permeability and the growth of eutrophic bacterial flora, and regulate the metabolism. Specific metabolites derived from the microbiota have also been identified in patients with MASLD, which could participate in the brain–gut cross-talk and in the pathogenesis of the disease [87].

**Table 2:** Machine learning and deep learning models applied to MASLD: expanded study characteristics.

Article	Study Design; Prospective or Retrospective	Population: Exact Age Range and Characteristics	Model-Development Sample; Validation Sample; Internal and External Validation	Outcome Definition and Reference Standard	Predictor Variables	Algorithm	Discrimination Metrics	Calibration	Interpretability/Explainability	Clinical Availability/Deployment Status	Pediatric Applicability
Das et al., 2021 [81]	Design: Retrospective digital image analysis of de-identified hepatic ultrasound images obtained as part of a cross-sectional study of pediatric NAFLD prevalence (Valleywise Health Medical Center, Phoenix, AZ, USA). Timing: Retrospective analysis of images collected in a prior cross-sectional study.	Children. Development set: 93 subjects with normal liver and 39 with confirmed NAFLD (*n* = 132). External validation set: 42 children. Exact age range, sex distribution and BMI not available.	Development: Unit of analysis is the region of interest, not the patient: 484 ROIs from 93 normal subjects and 260 ROIs from 39 subjects with NAFLD (744 ROIs from 132 children), each with 28 extracted texture features, used to develop, train and internally validate the model. Validation: External validation cohort of 42 children contributing 211 ROIs. Internal/external: Internal: Development plus internal validation on the 132-child dataset. External: Independent cohort of 42 children (211 ROIs).	NAFLD versus normal liver. The paper states “confirmed NAFLD” but does NOT specify the reference standard (histology, MRI-PDFF, elastography or ultrasound reading). This is a critical unreported item.	28 texture features extracted from a representative region of interest on hepatic ultrasound images using ImageJ and MAZDA image analysis software. Comparators: Hepatorenal index and hepatic echo-intensity attenuation index (pixel-intensity-based).	Ensemble ML model combining SVM, neural network and extreme gradient boosting. Multiple classification algorithms were evaluated.	Combined AUC for SVM, Multi-Layered Perceptron Neural Net and XGBoost 0.978 in the training set, 0.951 in the testing set, 0.969 in the validation set; AUC of 0.92 (95% CI, 0.91–0.94) when retesting in the external validation dataset.	Not reported	Hand-crafted texture features are inherently interpretable; no formal explainability method reported in the paper.	None. Research prototype.	Fully pediatric and the only pediatric imaging AI study in Table 2 with a separate external validation cohort. Ultrasound is widely available in pediatrics, which favors translation, but the age range and clinical characteristics of the children are not reported.
Wang et al., 2025 [82]	Design: Cross-sectional analysis of baseline data from the SCIENT cluster-randomized trial (ClinicalTrials.gov NCT05482165) conducted in six primary schools in Ningbo, China, September 2022 to June 2023. Timing: Cross-sectional analysis of prospectively collected trial baseline data.	161 grade 3 children aged 8–10 years overweight or obese by Chinese national standards (median age 8.5 y, IQR 8.3–8.8); 58/161 (36%) girls. Median BMI z-score 2.10 (non-MASLD) versus 2.72 (MASLD). 42/161 (26.1%) had MASLD. Exclusions: Heart disease, hypertension, diabetes, asthma, viral hepatitis, nephritis, secondary or drug-induced obesity, abnormal development, inability to take part in school sports, recent induced weight loss.	Development: 161 children with 42 outcome events; five-fold stratified cross-validation used for sequential forward feature selection and AUC estimation. Six predictors in the final model, i.e., approximately 7 events per predictor. Validation: Internal replication only: random 2:1 train-test split (*n* = 107/54) and a school-based split (4 schools, *n* = 110, for training; 2 schools, *n* = 51, for testing). No independent external cohort; the authors acknowledge that external validation in larger pediatric cohorts is still required. Internal/external: Internal: Five-fold stratified CV, 2:1 random split, school-based split. External: None.	MASLD defined as hepatic steatosis on vibration-controlled transient elastography (FibroScan, CAP ≥ 248 dB/m; median of 10 valid measurements, results with IQR/median >30% discarded) plus at least one cardiometabolic risk factor, which all participants met by virtue of overweight or obesity. Fibrosis defined as LSM ≥ 7.0 kPa (present in 1/42). Reference standard: VCTE, not histology or MRI-PDFF.	92 inflammation-related plasma proteins (Olink Explore 96 Inflammation panel, proximity extension assay); 82 retained after excluding proteins detected in <75% of samples; 20 significant on univariable analysis, 15 robust after adjustment for age, sex, BMI and school. Final six-protein ProScore panel: FGF-21, CDCP1, CD244, OPG, Flt3L, MCP-1. Comparators: 11 anthropometric/metabolic indices (WHtR, METS-IR, SPISE, PNFI, VAI, LAP, TyG, TyG-ALT, TyG-WC, TyG-WHtR, TyG-BMI) and a 9-SNP genetic risk score.	Six classifiers compared—logistic regression, RF, DT, SVM, XGBoost, LightGBM—with model-specific variable importance ranking followed by sequential forward feature selection. Logistic regression achieved the highest cross-validated AUC and was used to generate the ProScore (the published formula is a predicted probability).	Cross-validated AUC 0.836 Random 2:1 split test AUC 0.818; school-based split AUC 0.830. Sex-stratified: 0.907 (95% CI 0.830–0.984) in girls and 0.808 (0.717–0.898) in boys, DeLong *p* = 0.101. Stratified by genetic risk: 0.915 in the low-GRS group versus 0.800 in the high-GRS group, DeLong *p* = 0.003. Sensitivity analysis with CAP ≥ 270 dB/m: AUC 0.881 (0.807–0.955). Best comparator TyG-BMI 0.783; GRS 0.648. ProScore significantly exceeded every comparator (DeLong *p* < 0.05) and no combination improved on it.	Not assessed. No calibration plot, calibration slope or Brier score reported.	Model-specific variable importance (logistic and SVM coefficients, tree-based information gain), sequential forward selection, and GO/KEGG/Reactome pathway enrichment of the selected proteins. The final six-variable logistic model with a published formula is inherently interpretable. No SHAP or LIME.	None. The authors identify assay cost as a barrier and call for cross-platform validation using conventional assays (ELISA, targeted mass spectrometry) before translation.	High—fully pediatric (8–10 y) and restricted to children with overweight or obesity, exactly the target population of this review. Limitations for transfer: Very narrow age band, single Chinese city, pubertal status not assessed, VCTE reference standard, and dependence on a high-throughput proteomic platform that is not routinely available.
Li et al., 2025 [83]	Design: Cross-sectional single-center study, Department of Clinical Nutrition, Xi’an Children’s Hospital, Xi’an, China. Timing: Cross-sectional; timing of data collection relative to analysis not stated.	219 children with obesity: 79 with MASLD and 140 without. Children with MASLD had higher weight (59.8 vs. 45.5 kg), BMI (28.03 vs. 24.56 kg/m^2^), waist circumference (82.64 vs. 69.30 cm), WHtR (0.54 vs. 0.49), body fat mass (26.06 vs. 18.13 kg), visceral fat area (123.89 vs. 85.06 cm^2^), ALT (40 vs. 19 U/L) and AST (32 vs. 26 U/L). Associations were stronger in boys and in children with obesity duration >3 years.	Development: 219 children with 79 events. Validation: Internal validation, bootstrap-corrected calibration plot. No external validation.	MASLD in children with obesity.	Demographic, anthropometric, body-composition and biochemical parameters. Top features: Weight, waist circumference, body fat mass and ALT. ALT was additionally modeled as a mediator, accounting for 32.9–38.3% of the adiposity-MASLD association.	Multivariable logistic regression, RF, LASSO regression, mediation models and a nomogram.	AUC 0.861 for the combined model of weight, waist circumference, body fat mass and ALT, described by the authors as outperforming single-metric approaches.	Reported as a bootstrap-corrected calibration plot This is one of only three studies across both tables that assessed calibration.	Nomogram for individual risk stratification, RF feature ranking and formal mediation analysis quantifying the role of ALT.	None. A nomogram is proposed for personalized risk stratification but no tool or software is reported.	High—fully pediatric and restricted to children with obesity. All predictors (weight, waist circumference, bioimpedance-derived fat mass, ALT) are routinely obtainable in pediatric practice, making this the most immediately translatable model in Table 2. Limited by the single-center design, modest sample and absence of external validation.
Zhan et al., 2025 [84]	Design: Cross-sectional metabolomic biomarker study with group-balanced sampling, plus in vitro mechanistic work in mouse hepatocytes and liver organoids. Discovery participants recruited during hospital visits (Children’s Hospital, Zhejiang University School of Medicine, Hangzhou); validation participants sampled from schools. Timing: Cross-sectional; samples collected prospectively, models fitted retrospectively.	Discovery set *n* = 110: 30 normal weight, 30 healthy obesity, 30 obesity with MASL, 20 obesity with MASH. Mean age 11.65 (SD 2.20) y across groups, with a male predominance. Obesity by the Chinese Standard for Overweight and Obesity among School-Age Children and Adolescents; severity stratified by BMI z-score (overweight 1 to <2, mild obesity 2 to <3, severe obesity ≥3). Exclusions: Type 1 diabetes, chronic liver disease other than MASLD, drugs causing steatosis within 12 months. Validation set *n* = 112 children with obesity (37 healthy obesity, 56 MASL, 19 MASH), stratified—randomly selected from 336 children with obesity at two primary schools and one middle school.	Development: MASL versus healthy obesity model: 60 children (30 vs. 30). MASH versus MASL model: 50 children (20 vs. 30). Very small development samples. Validation: Independent validation cohort of 112 children with obesity recruited in a different setting (schools rather than hospital)—a genuine external validation in terms of setting and sampling. Internal/external: Internal: Model development in the discovery set. External: Independent 112-child validation cohort.	Hepatic steatosis diagnosed by proton magnetic resonance spectroscopy (1H-MRS), FibroScan OR liver ultrasound, i.e., the reference standard differed between participants. MASH defined non-invasively as imaging-confirmed steatosis plus persistent serum ALT >60 U/L for more than 3 months in the absence of secondary causes. No liver biopsy, which the authors justify on ethical grounds in children.	Untargeted plasma metabolomics (731 metabolites across four UPLC-MS/MS methods) plus clinical parameters. Final MASL-versus-healthy-obesity model: tryptophan (TRP), *N*-acetyltryptophan (AcTRP) and quinolinic acid (QA). Final MASH-versus-MASL model: *N*-acetyl-L-methionine, oxoglutaric acid, taurocholic acid, cysteine-glutathione disulphide and glycocholic acid.	Logistic regression for the MASL model and a neural network for the MASH model. Supporting analyses: PCA and OPLS-DA for exploratory profiling, fuzzy c-means (mfuzz) clustering of metabolite trajectories, and SHAP for interpretation.	Discovery set, MASL versus healthy obesity—single metabolites TRP 0.750, AcTRP 0.792, QA 0.693, versus clinical comparators ALT 0.745, BMI 0.752, UA 0.746; combined three-metabolite logistic model AUC 0.874 with sensitivity 86.7%, specificity 67.7%, accuracy 76.7%. MASH versus MASL-AST 0.944 and GGT 0.938 (ALT excluded as it is part of the MASH definition), TRP 0.743; combined five-metabolite neural network AUC 0.847 with sensitivity 75.0%, specificity 76.7%, accuracy 76%. Validation set: Performance is described only as “replicated” and “maintained”, shown graphically); no numerical auc or confidence interval is given in the text for the validation cohort.	Not assessed.	SHAP analysis applied to the final models, identifying AcTRP, TRP and QA as the dominant contributors; supported by mechanistic in vitro experiments showing that these metabolites promote hepatocyte lipid accumulation via oxidative stress.	None.	High—fully pediatric (mean 11.65 y), obesity-specific, and the only study in Table 2 with a genuinely independent validation cohort recruited in a different setting. Translation is limited by dependence on untargeted UPLC-MS/MS metabolomics and by a heterogeneous imaging reference standard.
Zöggeler et al., 2025 [85]	Design: Systematic review (MOOSE) with pooled individual-level reanalysis of raw shotgun metagenomic sequencing data from nine published studies plus one newly recruited cohort (Medical University of Innsbruck), followed by development of ML classifiers on the pooled data. Timing: Retrospective pooling of previously published datasets plus one prospectively recruited cohort.	413 children analyzed: 132 healthy controls, 58 with obesity, 153 with MASLD and 70 with MASH. Median ages—controls 9 y (IQR 5.7–12), obesity 13 y (9.6–14), MASLD 13 y (12–14.7; 62 missing), MASH 12 y (10–14). Median BMI—controls 16, obesity 30.2, MASLD 31.1, MASH 30.8. Female 45.5%, 46.6%, 31.8% and 24.3% respectively. Fibrosis in the MASH group: F0–F1 48.6%, F2 14.3%, F3–F4 8.6%, missing 28.6%. An additional adult MASLD cohort (*n* = 163) was included for pediatric-versus-adult comparison.	Development: Pooled pediatric data; group sizes vary by comparison (obesity *n* = 58 versus MASLD *n* = 153; MASLD *n* = 153 versus MASH *n* = 70), against hundreds of microbial species and metabolic pathway features. Validation: Internal only: 10-fold cross-validation with five repeats (R caret) and hyperparameter tuning. No external validation. Internal/external: Internal: Repeated 10-fold CV. External: None.	MASLD diagnosed by ultrasound, magnetic resonance imaging OR liver histology depending on the source study; MASH defined as additional biochemical OR biopsy-proven hepatic inflammation. Obesity defined as BMI at or above the 95th percentile. Studies including patients with T2DM were excluded. The reference standard therefore differs across participants.	Gut microbiome species abundance from shotgun metagenomic sequencing and MetaCyc metabolic pathway abundance (HUMAnN3), after MMUPHin batch-effect correction using study as the batch and disease state as a covariate.	RF and XGBoost (R caret), each with 10-fold cross-validation and five repeats and hyperparameter tuning; XGBoost shrinkage used to limit overfitting. Supporting analyses: Alpha and beta diversity, PERMANOVA, LEfSe/LDA, ALDEx2, MaAsLin2.	Taxonomy-based models—MASLD versus obesity: XGBoost AUROC 87% (95% CI 0.82–0.92), RF 85%; MASH versus MASLD: XGBoost and RF both 89% (0.84–0.94). Pathway-abundance models—MASLD versus obesity: XGBoost 81% (0.76–0.86), RF 79% (0.73–0.85); MASH versus MASLD: XGBoost 88% (0.83–0.93), RF 85% (0.79–0.91). Pediatric versus adult MASH: AUROC 97%.	Not assessed.	Ranked feature importance from both models, LEfSe/LDA differential abundance and pathway-level analyses identifying *Faecalibacterium prausnitzii, Prevotella copri*, Anaerobutyricum (*Eubacterium) hallii*, *Romboutsia timonensis* and *Intestinibacter bartlettii*, and the methanogenesis-from-acetate pathway. No SHAP.	None. Proposed as a potential fecal biomarker.	High in relevance—the largest pooled pediatric MASLD microbiome dataset available—but of limited practical applicability, since shotgun metagenomic sequencing is a research tool, the pooled cohorts span several countries and ethnicities, and the reference standard is heterogeneous.
Ji et al., 2022 [59]	Design: Cross-sectional targeted metabolomics study with ML, pooling participants from four parent studies at Gachon University Medical Center, Republic of Korea (healthy volunteers; an MR-based NAFLD study; a bariatric surgery cohort; and living liver transplant donors). Liver RNA sequencing performed in a subset. Timing: Retrospective pooled analysis of four parent studies.	86 adults aged 19–70 years: 25 healthy controls (mean age 35.2 y), 42 with NAFL (43.2 y) and 19 with NASH (41 y). Exclusions: Alcohol >20 g/day (women) or >30 g/day (men), other liver or biliary disease, drugs causing secondary steatosis within one year. Not obesity-defined. NOTE: Section 2.8 of the paper refers to 89 participants while the rest of the paper reports 86—an internal inconsistency.	Development: Pairwise 75%/25% splits: healthy versus NAFL 51 training/16 test; healthy versus NASH 34/10; NAFL versus NASH 47/14. Extremely small. Validation: Internal only: 10-fold cross-validation and leave-one-out cross-validation plus the 25% hold-out. No external validation; the authors themselves state that validation of the MetaNASH score in independent cohorts is warranted. Internal/external: Internal: 10-fold CV, LOOCV, 75/25 split. External: None.	NASH defined as either NAFLD activity score ≥ 4 on liver biopsy OR MRI-PDFF ≥ 16.1% together with MR elastography liver stiffness ≥ 3.8 kPa in participants without biopsy; NAFL as MRI-PDFF ≥ 5%; healthy controls as MRI-PDFF <5% with normal liver enzymes and no biopsy. Only 12 of 86 participants underwent liver biopsy, so for most the reference standard was an MR-based algorithm previously derived by the same investigators—which the authors acknowledge as the study’s main limitation.	79 plasma metabolites measured by GC-MS/MS and LC-MS/MS (amino acids, kynurenine pathway metabolites, nucleosides, organic acids, fatty acids). Eight selected by RF and multinomial logistic regression (glutamic acid, cis-aconitic acid, aspartic acid, isocitric acid, alpha-ketoglutaric acid, oxaloacetic acid, myristoleic acid, tyrosine); three retained by a recursive partitioning and regression tree (glutamic acid, isocitric acid, aspartic acid), combined into the MetaNASH score = log10(aspartic acid^1^ × isocitric acid^2^ × glutamic acid^4^).	RF (primary), multinomial logistic regression, recursive partitioning and regression tree, with PLS-DA for exploratory multivariate analysis.	RF classifiers—NAFL versus healthy controls (8 features) AUROC 0.900 (95% CI 0.854–0.947); NASH versus healthy controls all classifiers >0.960, best 0.990 with 4 features; NASH versus NAFL best 0.849 with 4 features. MetaNASH score for NASH discrimination: AUROC 0.877 at a cut-off of 4.55, computed in the WHOLE cohort WITHOUT any training/test separation, i.e., an apparent (optimistic) estimate.	Not assessed.	RF variable importance, an explicit DT, multinomial logistic regression coefficients, and integration of metabolomics with liver RNA-seq to build a metabolic map linking plasma metabolites to hepatic enzyme expression. The final MetaNASH score is a closed-form, fully transparent formula.	None.	None. Eligibility was restricted to ages 19–70 years; no pediatric data.
Noureddin et al., 2022 [60]	Design: Retrospective cross-sectional cohort analysis of the nationally representative NHANES 2017–2018 survey (United States). Timing: Retrospective analysis of cross-sectional survey data.	Adults aged ≥ 20 years with valid reproducible transient elastography (>10 measurements, IQR <30% of the median). From 5494 with a completed examination, 4471 met age and validity criteria; after excluding 640 with high alcohol consumption, viral hepatitis or HIV, 3831 entered the ML analysis (1226 with NAFLD, 2605 without). A T2DM subpopulation of 908 included 468 with NAFLD. Known over-representation of participants aged ≥ 60 y and of African American and Hispanic participants. Not obesity-selected.	Development: 2874 in the training set (75%), with 10-fold cross-validation and five replications for hyperparameter tuning. Over 100 candidate features. Validation: 957 in the internal test set (25%). No external validation. Internal/external: Internal: 75/25 split with 10-fold CV × 5 repeats. External: None.	NAFLD defined as controlled attenuation parameter ≥ 302 dB/m (Youden-optimal threshold) on FibroScan; fibrosis staged by VCTE liver stiffness (F0–F1 ≤ 8.2, F2 ≤ 9.7, F3 ≤ 13.6, F4 > 13.6 kPa). No participant had biopsy confirmation, and other causes of liver disease such as autoimmune hepatitis, primary biliary cholangitis and haemochromatosis could not be excluded; alcohol intake was self-reported and may be underestimated. Both limitations are acknowledged by the authors.	Over 100 features: demographics (age, race/ethnicity, sex, marital status, education), clinical characteristics (BMI, waist circumference, blood pressure), laboratory values (HbA1c, AST, ALT, alkaline phosphatase, bilirubin, HDL) and comorbidities (diabetes, hypertension). Significant predictors on logistic regression: male sex, HbA1c, age, BMI, waist circumference, AST, alkaline phosphatase, diastolic blood pressure and HDL.	Six models compared: Logistic regression, elastic net, conditional single-classification tree, RF, SVM and neural network. Logistic regression selected as the model of choice for simplicity and interpretability given equivalent performance.	Test-set AUROC: elastic net 0.84 (0.81–0.86), logistic regression 0.83 (0.81–0.86), RF 0.83 (0.80–0.86), SVM 0.83 (0.80–0.85), neural network 0.83 (0.80–0.85), classification tree 0.79 (0.76–0.82). Accuracy 0.75–0.79; sensitivity 0.52–0.71; specificity 0.78–0.90; PPV 0.60–0.72; NPV 0.80–0.85.	Not assessed.	Logistic regression and elastic net explicitly chosen as the “interpretable” model classes, with odds ratios reported for individual predictors. No SHAP or LIME.	None. Proposed for identifying candidates for further assessment across large datasets.	None. Adults aged ≥ 20 years, with over-representation of participants aged ≥ 60 y.
Razmpour et al., 2023 [61]	Design: Cross-sectional study with volunteer recruitment by advertisement on university clinic notice boards and by telephone or email, in Khorasan Razavi (east) and Hormozgan (south) provinces, Iran. Timing: Cross-sectional with prospective recruitment of volunteers; retrospective modeling.	593 recruited, 80 excluded, 513 analyzed. The Methods state participants aged above 13 years, but Table 1 of the paper gives an age range of 9–74 years (mean 37.04, SD 15.44) and guardians consented for participants under 18, so a minority of children were included. Mean weight 77.26 (SD 17.31) kg; mean BMI 28.15 (SD 4.89) kg/m^2^, range 15–52; 13.3% diabetic. Sex reported as 240 male/273 female in the text but 238 male/270 female in Table 1 of the original paper (*n* = 508), an internal inconsistency with the stated 513. Not obesity-selected. Exclusions: Underlying liver disease, several drug classes, alcohol use more than twice weekly, cancer in the past year, surgery in the past 6 months, pregnancy.	Development: 513 participants split into train and test sets (proportions NOT stated); each classifier trained and evaluated 50 times with results averaged. Validation: Internal only. The authors state that future studies with larger samples could “allocate separate validation sets”, implying no dedicated held-out validation set. No external validation. Internal/external: Internal: repeated train/test evaluation over 50 runs. External: None.	Hepatic steatosis and fibrosis staged by transient elastography (FibroScan): steatosis grade 3 at CAP ≥ 292 dB/m; fibrosis F0 < 6.2, F1 6.2–7.6, F2 7.6–8.8, F3 8.8–11.8, F4 ≥ 11.8 kPa. Three labels modeled: fatty liver present versus absent, steatosis stage, fibrosis stage. No histological confirmation; the authors acknowledge that this is not the gold standard.	Body composition and anthropometric indices. Most important features for fatty liver: abdomen circumference (importance 0.061), waist circumference (0.061), trunk fat (0.056), chest circumference (0.054) and BMI (0.053); similar rankings for steatosis and fibrosis staging, with sex-specific analyses reported.	Eight classifiers compared in scikit-learn: k-nearest neighbors, SVM, radial basis function SVM, Gaussian process, RF, neural network, AdaBoost and naïve Bayes, with PCA for feature extraction. RF performed best.	RF—fatty liver (any stage): accuracy 0.82, AUC 0.84; steatosis stage: accuracy 0.52, AUC 0.69; fibrosis stage: accuracy 0.57, AUC 0.58.	Not assessed.	Feature importance values reported for each outcome and separately by sex. No SHAP or LIME.	None. Proposed as a low-cost screening decision-support approach for primary care and remote settings.	Minimal. Although the reported age range begins at 9 years and minors were enrolled with guardian consent, the mean age is 37 years and no pediatric subgroup analysis was performed. The current Limitations entry “non pediatric data included” is therefore not strictly accurate; the accurate statement is that a small unspecified number of minors were included without any pediatric-specific analysis.
Huang et al., 2023 [62]	Design: prospective 5-year cohort study (baseline health checkups in 2010, follow-up to 2015), Zhenhai Lianhua Hospital, Ningbo, China; reported in accordance with STROBE. The only prospectively designed model-development study in either table. Timing: prospective cohort with 5-year follow-up.	From 17,611 attendees of the 2010 annual health checkup, 6196 adults without NAFLD were enrolled after excluding those without liver ultrasound, with existing liver disease, with alcohol intake >140 g/week (men) or >70 g/week (women), or lost to follow-up. Incident NAFLD occurred in 1155 (18.64%) over 5 years, of whom 941 (81.5%) were men. Among incident cases, 434 (37.6%) were lean (BMI < 24), 603 (52.2%) overweight (24 to <28) and 118 (10.2%) obese (≥28 kg/m^2^)—i.e., the cohort is NOT obesity-selected and most incident cases were not obese.	Development: Training and internal validation split 7:3; 10-fold cross-validation within the training set; hyperparameter optimization; SMOTE applied for class imbalance. 11 predictors selected, with the authors stating explicit compliance with the 10-events-per-variable rule. Validation: Internal validation set (30% split) and an external validation set consisting of the 2015–2020 follow-up population—a temporal external validation at the same center. Internal/external: Internal: 7:3 split plus 10-fold CV. External: Temporal external validation using the 2015–2020 follow-up population.	Incident NAFLD over 5 years, diagnosed on abdominal ultrasound (Toshiba Medical Systems) read independently by experienced ultrasonographers, with exclusion of excessive alcohol intake and other etiologies. No histological confirmation; the authors list ultrasound-based diagnosis as their first limitation.	11 characteristic predictors screened by XGBoost recursive feature elimination combined with LASSO. The published nomogram uses age, BMI, waist circumference, white blood cell count, red blood cell count, ALT, gamma-glutamyl transpeptidase, uric acid, triglycerides, HDL and apolipoprotein-B. Restricted cubic splines used to characterize dose–response relationships.	Six models compared: Logistic regression, DT, SVM, RF, CatBoost and XGBoost. Logistic regression selected as the final model.	Training set (10-fold CV): CatBoost AUROC 0.810 (95% CI 0.768–0.852), RF 0.800 (0.762–0.838). Internal validation: Logistic regression 0.778 (0.759–0.794). EXTERNAL validation: Logistic regression 0.806 (0.788–0.821), with accuracy 0.801, precision 0.766, F1 0.648 and recall 0.628.	Asessed-calibration curves and Brier scores reported for both the internal and external validation sets; XGBoost showed the best calibration (Brier score 0.181 internally and 0.191 externally). One of only three studies in the whole review to report calibration.	Dynamic nomogram with a worked individual example, logistic regression coefficients, restricted cubic splines, and transparent XGBoost-RFE plus LASSO feature screening. The final model is inherently interpretable. No SHAP.	A web-based risk calculator was developed and published to support clinical use—the only model in either table with a publicly deployed tool. It is not reported as a regulated medical device and has not undergone prospective impact evaluation.	None. Adult health-checkup population; the authors note that applicability to other ethnic groups also remains unvalidated.
Qin et al., 2023 [63]	Design: Cross-sectional diagnostic/screening study using electronic medical record data from attendees of the annual health examination at Guilin People’s Hospital, China, January to December 2021. Timing: Retrospective analysis of a cross-sectional health examination dataset.	14,439 adults aged ≥ 18 years: 4411 with NAFLD (30.5%) and 10,028 without. NAFLD group mean age 48.4 (SD 13.4) y, 76.6% male, mean BMI 27.2 (SD 3.2); non-NAFLD group 45.9 (14.8) y, 53.8% male, BMI 23.5 (3.0). Exclusions: alcohol > 210 g/week (men) or >140 g/week (women); viral, autoimmune or drug-induced liver disease and other chronic or secondary liver disease; acute or chronic infection; pregnancy or lactation; psychiatric disorders; malignancy. Not obesity-selected.	Development: Random 7:3 split, approximately 10,107 in the training set. Grid search with 10-fold cross-validation for hyperparameters. z-score standardization fitted on the training set and applied unchanged to the test set. Validation: Internal only: Approximately 4332 in the test set. No external validation. Internal/external: Internal: 7:3 split with grid search and 10-fold CV. External: None.	NAFLD diagnosed by color Doppler ultrasound (GE LOGIQ, 3.5 MHz probe) by operators with at least 5 years of experience, with secondary causes excluded. No histological confirmation.	Age, sex, BMI, systolic and diastolic blood pressure, complete blood count, liver function panel (bilirubins, ALT, AST, AST/ALT ratio, GGT, ALP, total protein, albumin, globulin, albumin/globulin ratio) and lipid panel (triglycerides, total cholesterol, HDL, LDL, VLDL), all drawn from the electronic medical record. Most important features across the tree-based models: BMI, triglycerides, ALT and the AST/ALT ratio (plus VLDL-C for RF).	DT (CART, gini), RF, XGBoost and SVM, implemented in Python/scikit-learn.	Test set—SVM: accuracy 0.801, PPV 0.795, F1 0.795, kappa 0.508, AUPRC 0.712, AUROC 0.850; RF: AUROC 0.852 (the highest of any model), accuracy 0.789, PPV 0.782, F1 0.782, kappa 0.478, AUPRC 0.708; XGBoost: AUROC 0.833, accuracy 0.781; DT: AUROC 0.820, accuracy 0.765. All with 95% confidence intervals. The SVM AUROC is 0.850.	Not assessed.	Feature importance reported for every classifier, including an explicit DT structure and XGBoost gain rankings. The authors deliberately favored a small predictor set to keep the tool usable in primary care. No SHAP.	None. Proposed as a screening aid for physicians and primary care doctors.	None. Adults aged ≥ 18 years.
Rhyou and Yoo, 2021 [73]	Design: Retrospective deep learning image classification study on B-mode liver ultrasound images. The unit of analysis is the IMAGE, not the patient. Timing: Retrospective.	The number of individual patients, their age, sex, BMI, obesity status, indication for scanning and clinical setting are all absent. Images were pooled from two sources: a Samsung ACUSON Sequoia 512 dataset and the publicly available Byra dataset acquired on a GE Vivid E9.	Development: Ultrasound images split 6:2:2 into training, validation and test sets at the IMAGE level. Class labels: normal, mild, moderate, severe steatosis. Validation: Internal validation and test partitions of the same pooled image dataset. No external validation—the public Byra dataset was merged into the training/validation/test pool rather than held out as an independent test set. Internal/external: Internal: 6:2:2 image-level split. External: None.	Steatosis grade (normal, mild, moderate, severe) as previously annotated by medical experts on the ultrasound images. The reference standard is therefore expert visual reading of the SAME image supplied to the model; no histology, MRI-PDFF or biochemical confirmation. The number of readers, their independence and inter-reader agreement are not reported.	Ultrasound image pixel data from the liver–kidney region, after resizing to 960 × 720 px, removal of metadata and black borders, and histogram equalization.	Cascade of three deep neural networks: DeepLabv3+ with transfer learning for semantic segmentation of the liver–kidney area; Inception v3 (transfer-learned) for classifying parasagittal versus non-parasagittal images and for ring detection; and SteatosisNet for grading disease severity.	Sensitivity 99.78%, specificity 100%, PPV 100%, NPV 99.83%, diagnostic accuracy 99.91%; parasagittal detection accuracy 99.90%. No confidence intervals. These values are implausibly high for ultrasound-based steatosis assessment and are characteristic of image-level rather than patient-level data partitioning.	Not assessed.	Segmentation masks and cropped regions of interest can be visually inspected, providing a degree of transparency about which anatomy drives the prediction. No formal explainability method.	None.	None, and not assessable—no patient ages are reported anywhere in the paper. Pediatric liver ultrasound differs in acquisition and in normal appearances, so transfer to children cannot be assumed.
Zhou et al., 2025 [67]	Design: Cross-sectional analysis of NHANES 2017–2018 using the survey’s stratified multistage probability design with sampling weights applied throughout. Timing: Retrospective analysis of cross-sectional survey data.	From 9254 initial participants, 3306 were excluded for missing controlled attenuation parameter data and 894 for not meeting MASLD diagnostic eligibility, leaving 5054 US adults aged ≥ 18 years. Median age by visceral adiposity index tertile 44, 47 and 50 years; median BMI 25.9, 28.7 and 31.3 kg/m^2^; sex distribution balanced. Not obesity-selected.	Development: Stratified 7:3 training/test split; 11 predictors selected by LASSO regression with 10-fold cross-validation using the lambda.1se criterion. Validation: Internal only (30% test partition). No external validation. Internal/external: Internal: Stratified 7:3 split with LASSO/10-fold CV for predictor selection. External: None.	MASLD defined by controlled attenuation parameter-based hepatic steatosis on transient elastography together with cardiometabolic criteria. No histological confirmation.	11 LASSO-selected predictors: visceral adiposity index, marital status, hypertension, diabetes, sex, age, race, platelet count, albumin, AST and ALT. Comparators: Seven established non-invasive scores.	Eight ML models compared (RF, SVM, generalized linear model, gradient boosting machine, k-nearest neighbors, neural network, DT) plus a nomogram.	RF AUC 0.869, gradient boosting machine 0.868, generalized linear model 0.852, neural network 0.504 (i.e., no better than chance, unexplained). Non-invasive scores: lipid accumulation product 0.834, fatty liver index 0.833, hepatic steatosis index 0.799, visceral adiposity index 0.736, TyG index 0.732, NAFLD fibrosis score 0.606, FIB-4 0.553. VAI itself showed a strong dose–response association with MASLD (adjusted OR for tertile 3 versus 1: 7.08, 95% CI 4.35–11.5; *p* for trend 0.003). The authors’ own conclusion is that the simple LAP and FLI scores, not the machine learning models, offer the best balance of accuracy and clinical practicality.	Not reported. A nomogram is presented without an accompanying calibration curve.	LASSO-selected predictor set, a nomogram, and explicit benchmarking against seven interpretable clinical scores. No SHAP.	None. The authors explicitly note that ML models face translational barriers from algorithmic complexity and resource demands, whereas LAP and FLI provide immediate clinical utility.	None. Adults aged ≥ 18 years.

Abbreviations used in the Tables: AcTRP, *N*-acetyltryptophan; ADA, American Diabetes Association; AdaBoost, adaptive boosting; ALP, alkaline phosphatase; ALT, alanine aminotransferase; ANN, artificial neural network; Apo-B, apolipoprotein B; AST, aspartate aminotransferase; AUC, area under the curve; AUPRC, area under the precision-recall curve; AUROC, area under the receiver operating characteristic curve; BFM, body fat mass; BMI, body mass index; BRI, body roundness index; CAP, controlled attenuation parameter; CART, classification and regression tree; CatBoost, categorical boosting; CI, confidence interval; CKD, chronic kidney disease; CNN, convolutional neural network; CT, computed tomography; CV, cross-validation; DKD, diabetic kidney disease; DL, deep learning; DT, decision tree; ELISA, enzyme-linked immunosorbent assay; EMR, electronic medical record; EPV, events per variable; FFQ, food frequency questionnaire; FIB-4, fibrosis-4 index; FLI, fatty liver index; FPG, fasting plasma glucose; GB, gradient boosting; GBDT, gradient boosting decision tree; GBM, gradient boosting machine; GC-MS/MS, gas chromatography–tandem mass spectrometry; GGT, gamma-glutamyl transferase; GRS, genetic risk score; HbA1c, glycated hemoglobin; HDL, high-density lipoprotein; HOMA-B, homeostasis model assessment of beta-cell function; HOMA-IR, homeostasis model assessment of insulin resistance; HSI, hepatic steatosis index; HU, Hounsfield unit; IDI, integrated discrimination improvement; IQR, interquartile range; ISPAD, International Society for Pediatric and Adolescent Diabetes; KNN, k-nearest neighbors; LAP, lipid accumulation product; LASSO, least absolute shrinkage and selection operator; LC-MS/MS, liquid chromatography–tandem mass spectrometry; LDL, low-density lipoprotein; LEfSe, linear discriminant analysis effect size; LightGBM, light gradient boosting machine; LOOCV, leave-one-out cross-validation; LR, logistic regression; LSM, liver stiffness measurement; MASH, metabolic dysfunction-associated steatohepatitis; MASL, metabolic dysfunction-associated steatotic liver; MASLD, metabolic dysfunction-associated steatotic liver disease; MCC, Matthews correlation coefficient; METS-IR, metabolic score for insulin resistance; ML, machine learning; MLP, multilayer perceptron; MLR, multinomial logistic regression; MODY, maturity-onset diabetes of the young; MRE, magnetic resonance elastography; MRI-PDFF, magnetic resonance imaging proton density fat fraction; 1H-MRS, proton magnetic resonance spectroscopy; mRMR, minimum redundancy maximum relevance; NAFL, non-alcoholic fatty liver; NAFLD, non-alcoholic fatty liver disease; NAS, NAFLD activity score; NASH, non-alcoholic steatohepatitis; NB, naïve Bayes; NFS, NAFLD fibrosis score; NHANES, National Health and Nutrition Examination Survey; NNET, neural network; NPV, negative predictive value; NRI, net reclassification index; OGTT, oral glucose tolerance test; OPLS-DA, orthogonal partial least squares discriminant analysis; OPG, osteoprotegerin; OR, odds ratio; PCA, principal component analysis; PLS-DA, partial least squares discriminant analysis; PNFI, pediatric NAFLD fibrosis index; PPV, positive predictive value; PROBAST, Prediction model Risk Of Bias ASsessment Tool; QA, quinolinic acid; RCS, restricted cubic spline; RF, random forest; RFE, recursive feature elimination; ROC, receiver operating characteristic; ROI, region of interest; SD, standard deviation; SHAP, Shapley additive explanations; SMOTE, synthetic minority oversampling technique; SNP, single nucleotide polymorphism; SPISE, single-point insulin sensitivity estimator; SVM, support vector machine; T2DM, type 2 diabetes mellitus; TRP, tryptophan; TyG, triglyceride–glucose index; UMAP, uniform manifold approximation and projection; UPLC-MS/MS, ultra-performance liquid chromatography–tandem mass spectrometry; VAI, visceral adiposity index; VCTE, vibration-controlled transient elastography; WC, waist circumference; WHtR, waist-to-height ratio; XGBoost, extreme gradient boosting.

### 3.3. Limitations

A challenge in the implementation of artificial intelligence (AI) in pediatric obesity is ensuring that algorithms are equitable, safe, and applicable across diverse populations. Many AI models are developed using datasets derived from specific geographic regions, healthcare systems, or academic centers, limiting their generalizability and increasing the risk of dataset shift when applied to different countries or clinical settings. Socioeconomic and geographic biases may further compromise model performance, as disadvantaged populations are frequently under-represented in training datasets. Unequal access to healthcare resources—including wearable devices, advanced imaging, laboratory investigations, and multi-omics technologies—may exacerbate these disparities, reducing the applicability of AI-driven approaches in low-resource settings and potentially widening existing health inequalities. Moreover, differences in predictive performance according to sex, ethnicity, and age have rarely been systematically evaluated, despite evidence that obesity-related risk factors, disease trajectories, and healthcare access vary substantially across these subgroups. Future studies should therefore incorporate subgroup-specific performance analyses and fairness assessments to identify and mitigate algorithmic bias before clinical implementation.

The pediatric setting also raises unique ethical and practical considerations. AI systems often require continuous collection of sensitive health data over prolonged periods, making long-term monitoring, data governance, and safety essential concerns. The use of children’s data requires robust procedures for parental informed consent and, whenever developmentally appropriate, child assent, while ensuring compliance with evolving privacy regulations. Because children undergo rapid physiological, behavioral, and developmental changes, predictive models must also be periodically updated and revalidated to maintain their accuracy over time. Ultimately, unless these methodological, ethical, and equity-related challenges are adequately addressed, AI systems risk reproducing or even amplifying existing disparities in healthcare delivery rather than reducing them. Ensuring representative datasets, transparent reporting, external validation across heterogeneous populations, and rigorous evaluation of algorithmic fairness should therefore be considered fundamental prerequisites for the responsible translation of AI into routine pediatric obesity care [20,25,28].

## 4. Discussion

Childhood obesity is a complex multifactorial disease that remains a major therapeutic challenge, particularly because high treatment dropout rates hinder both long-term management and the early detection of obesity-related complications. Thanks to AI and through GWASs [15,29], studies about the epigenome [5], metabolomics [57], the transcriptome [56], and the microbiome [22,24,54], it has been possible to broaden the spectrum of factors predisposing the onset of obesity in childhood. Thanks to ML, metabolites, intestinal bacteria, neurotransmitters and brain regions [22] involved in the onset of obesity have been identified, as well as SNPs in genes involved in lipid and carbohydrate metabolism, energy homeostasis and the hunger–satiety mechanism that predispose an increase in BMI [3,4]. It is also possible to integrate ecological and demographic data [21,30], using ML and DL to estimate the risk of obesity in a given population, depending on the child’s environment. Thus, it is possible to estimate the risk of developing complications for an individual based on multi-omics integration [20]. Among the most frequent complications, already present in pediatric age, are MS, prediabetes, T2DM, MASLD, all united by metainflammation and by insulin resistance [11]. It is known that the probability of developing these complications depends on the duration of obesity, however several studies have demonstrated a genetic and epigenetic susceptibility underlying this risk [25,27,28]. Although ML has been widely applied to the prediction, stratification, and management of T2DM, evidence in pediatric populations remains very limited. Overall, the pediatric evidence supports only a limited number of AI models. Most predictive algorithms—including SVM, RF, ANN, and XGBoost—have been extensively evaluated in adults, whereas pediatric studies are generally restricted to single-center cohorts with relatively small sample sizes. Consequently, current evidence supports the feasibility of these approaches in children rather than their clinical generalizability. To date, only the study by Yang et al. developed an ML model specifically to predict T2DM onset in children with obesity. In a cohort of 292 obese children, eight ML algorithms were compared, with the SVM achieving the best performance. The final model included eight predictors—BMI, creatinine, prealbumin, thyrotropin, total thyroxine, free thyroxine, glycated hemoglobin (HbA1c), and plasma glucose 180 min after an oral glucose tolerance test—highlighting the value of combining metabolic and endocrine markers. However, the model was developed in a relatively small cohort, and neither external validation nor calibration was reported, limiting its generalizability and current clinical applicability [41]. In contrast, numerous studies have evaluated ML models in adults with obesity and T2DM. Among the available approaches, RF, GB, SVM, ANN, LR, and DT have consistently shown good predictive performance, with RF and GB emerging as particularly promising because of their ability to model complex nonlinear relationships. The best-predicted outcomes include incident T2DM, prediabetes, diabetes-related complications, postprandial glycemic responses, and hypoglycemia. A systematic review by Nomura et al. reported that GB-, RF-, and LR-based models can predict T2DM up to five years before diagnosis [43]. Other studies have shown that ANN effectively identifies individuals with prediabetes and T2DM, while RF- and DT-based models accurately associate both traditional and emerging risk factors with disease onset. ML has also identified less conventional predictors, such as short sleep duration, high sodium intake, psychological stress, environmental pollution, and urban residence. Beyond risk prediction, ML has contributed to identifying biomarkers of β-cell dysfunction, including depletion of mature insulin-producing cells and inflammatory remodeling of pancreatic cell populations. Notably, several predictors recurred consistently across independent studies, including BMI, waist circumference, fasting glucose, HbA1c, triglycerides, and age. Their repeated identification across different ML algorithms suggests that these variables represent robust predictors rather than model-specific findings.

ML can be useful for predicting diabetes complications. Clustering approaches identified clinically meaningful T2DM subgroups with distinct complication profiles, including severe insulin deficiency associated with diabetic retinopathy and severe insulin resistance associated with diabetic nephropathy. Similar models have consistently identified early-onset T2DM phenotypes with poor glycemic control and increased microvascular risk. Beyond prediction, ML has demonstrated clinical potential in precision nutrition and diabetes management. GB-based algorithms integrating clinical, dietary, lifestyle, and gut microbiota data accurately predicted individualized postprandial glycemic responses, enabling personalized dietary interventions that improved glycemic control. In clinical practice, decision-support systems such as AdvisorPro for insulin dose adjustment and Guardian for hypoglycemia prediction illustrate how ML can be translated into patient care [43,51].

Despite these encouraging results, relatively few studies have performed external validation, and calibration is infrequently reported despite being essential for reliable risk prediction. Across the reviewed literature, external validation remains the exception rather than the rule. Apart from the ultrasound-based MASLD model externally validated in an independent pediatric cohort [81], no pediatric prediction model identified in this review underwent robust multi-center external validation. Calibration, which determines whether predicted risks correspond to observed outcomes, was rarely evaluated. Consequently, even models showing excellent discrimination cannot yet be considered reliable for individual clinical decision-making. Consequently, although some ML applications have reached clinical implementation, most prediction models remain retrospective and require prospective validation before widespread adoption. The greatest evidence gap remains in children. Compared with the extensive adult literature, pediatric evidence is limited to a single predictive study, with no largemulti-centerr cohorts, external validation, calibration analyses, or implementation studies. Future research should focus on developing and validating pediatric-specific models that account for growth, pubertal development, and the unique metabolic characteristics of children with obesity.

AI has substantially expanded the understanding of MASLD by integrating transcriptomic, lipidomic, metabolomic, microbiome, and clinical data. ML has identified novel genes, including AXUD1, FOSB, GADD45B, and SOCS2, metabolomic biomarkers such as glutamic acid, isocitric acid, and C4BPA, and environmental factors including pesticide exposure that may contribute to disease development [56,57,58]. These approaches have also enabled large-scale electronic medical record analyses for disease surveillance, risk stratification, prognosis, and personalized therapeutic approaches, including prediction of dietary and pharmacological responses.

Among predictive models, RF, SVM, and XGBoost consistently demonstrate the best performance for identifying MASLD in individuals with obesity. RF- and SVM-based models have been validated for predicting MASLD using clinical and demographic variables, while an XGBoost model developed by Zhang et al. [65] achieved high predictive accuracy using clinical and biochemical data, identifying waist circumference as one of the strongest predictors. Other ML models identified the roundness index, triglyceride–glucose index, and visceral adiposity index as effective predictors of MASLD. Across studies, the most recurrent predictors include BMI, waist circumference, triglycerides, liver enzymes (particularly ALT), age, sex, and features of metabolic syndrome. Dietary and lifestyle factors—including consumption of high-glycemic foods, low flavonoid and vitamin D intake, poor psychological well-being, and low self-perceived health status—have also emerged as important predictors. BMI and triglycerides have additionally been associated with progression from steatosis to MASH. Beyond disease prediction, ML has demonstrated value in prognostic assessment. ML models outperform conventional fibrosis scores such as FIB-4 by approximately 10% in identifying advanced liver fibrosis using clinical, anthropometric, and biochemical variables. Additional applications include prediction of hepatocellular carcinoma risk and cardiovascular risk stratification through CART models. DL, particularly CNNs, has shown excellent performance in imaging-based diagnosis. CNNs accurately detect hepatic steatosis on ultrasound, quantify liver fat on computed tomography, characterize histological features of MASLD and MASH, and classify fibrosis stages from biopsy and elastography images. Integrating radiomic features with circulating biomarkers and metabolomic data further improves diagnostic performance, highlighting the potential of multimodal AI approaches.

Despite these advances, clinical implementation remains limited. Although several models have undergone internal validation and a few have been externally validated, external validation across diverse populations is still uncommon, and calibration is rarely reported. Consequently, most models remain research tools rather than clinically deployable decision-support systems. The evidence gap is particularly evident in children. Compared with the adult literature, relatively few pediatric studies have included large cohorts or independent external validation. One ML model combining SVM, neural networks, and XGBoost demonstrated good diagnostic performance for ultrasound-based MASLD detection in 132 children and was externally validated in an independent cohort [81]. Other pediatric studies have identified circulating inflammatory proteins (as the ProScore), anthropometric measures (BMI, waist circumference, fat mass, and ALT), metabolomic biomarkers such as tryptophan derivatives, and gut microbiome signatures as promising predictors of MASLD. ML has also identified microbiota profiles associated with disease progression in adults, including *Prevotella copri*, which promotes hepatic lipid accumulation, and reduced abundance of *Eubacterium hallii*, suggesting a potential protective role. Nevertheless, pediatric evidence remains limited, with few prospective, multi-center studies, little assessment of calibration, and limited evaluation of real-world clinical utility. Larger externally validated pediatric cohorts integrating clinical, imaging, and multi-omics data are needed before AI-based MASLD prediction models can be routinely implemented in pediatric practice. Among the reviewed approaches, AI-assisted imaging for MASLD detection, ML-based prediction of postprandial glycemic responses, and clinical decision-support systems for diabetes management currently appear closest to clinical translation, owing to their validation in prospective clinical settings and integration into decision-support workflows [51,81]. Conversely, multi-omics prediction models integrating genomics, transcriptomics, metabolomics, and microbiome data remain largely exploratory. Although biologically informative, these approaches are limited by small sample sizes, high dimensionality, lack of standardization, and insufficient external validation. Importantly, higher predictive performance does not necessarily imply superiority over conventional statistical approaches. In several studies, logistic regression achieved discrimination comparable to more complex ML algorithms, particularly when based on routinely available clinical variables. Therefore, the added clinical value of AI should not be assumed solely from higher AUC values but should also consider calibration, interpretability, implementation costs, and prospective clinical impact. At present, no AI-based prediction model can be recommended for routine pediatric obesity care. Although several algorithms demonstrate promising retrospective performance, none has accumulated sufficient evidence regarding external validation, calibration, prospective evaluation, and clinical impact to support routine implementation. A recurring finding across all obesity-related complications is the distinction between statistical discrimination and clinical usefulness. Many AI models achieve high discrimination in retrospective datasets; however, discrimination alone is insufficient to justify clinical implementation. Robust calibration, external validation across heterogeneous pediatric populations, prospective impact studies, and demonstration of incremental benefit over existing clinical prediction tools remain largely absent. Until these requirements are fulfilled, AI should be considered a promising adjunct to clinical decision-making rather than a replacement for established pediatric assessment. These findings should also be interpreted in light of the methodological limitations discussed in the following section, including small pediatric cohorts, class imbalance, heterogeneous outcome definitions, missing data, limited model transparency, and the lack of standardized reporting, all of which currently restrict the translation of AI models into routine pediatric practice. Furthermore, very few studies have evaluated whether AI-assisted decision-making improves clinician performance, patient outcomes, or cost-effectiveness compared with standard care. Such impact analyses represent a crucial step before routine clinical implementation.

## 5. Conclusions

ML and DL have made significant contributions to the diagnosis, risk stratification for complications, prognosis, and treatment of childhood obesity. By integrating multimodal data—including clinical, biochemical, social, behavioral, ecological, genetic, epigenetic, radiological, and histological information—it is possible to develop a comprehensive profile for each patient. This phenotyping enables personalized risk estimation for disease progression and obesity-related complications, supporting targeted interventions tailored to the patient’s clinical phenotype and representing a promising step toward precision medicine. AI-based software may also optimize lifestyle interventions, facilitate intelligent monitoring of complications, and promote greater patient engagement and awareness. Despite these promising developments, several important limitations currently prevent the routine clinical implementation of most AI models. The available evidence for pediatric T2DM prediction remains very limited, and prediction models developed in adults cannot be directly transferred to children because of substantial differences in disease pathophysiology, clinical presentation, and developmental factors. Among obesity-related complications, externally validated pediatric models for MASLD, particularly those based on imaging or routinely available clinical variables, appear to be the most promising; however, their performance and generalizability remain insufficient to support routine clinical use. Similarly, emerging multi-omics and microbiome-based approaches offer valuable insights into disease mechanisms and may improve future predictive performance, but they are still largely exploratory and require further validation before clinical translation. Several methodological challenges also need to be addressed. Most published models have been developed and evaluated using retrospective datasets from limited populations, with insufficient external validation across diverse healthcare settings. In addition to external validation, future studies should systematically assess model calibration, fairness across clinically relevant demographic and socioeconomic subgroups, and prospective clinical impact to determine whether AI-assisted decision-making improves patient outcomes in real-world practice. Until these requirements are met, most of the reviewed models should not be used for routine individual clinical decision-making but rather considered as promising research tools. Beyond methodological issues, AI implementation presents important practical and ethical challenges. AI systems rely on large, high-quality datasets, requiring substantial investments in data collection, storage, integration, maintenance, and standardization. The heterogeneous data used for model development must be carefully curated and validated by expert clinicians before analysis. Moreover, AI raises concerns regarding patient privacy, data security, informed consent, algorithmic transparency, legal accountability, and the potential to exacerbate health inequalities due to unequal access to digital technologies and varying levels of digital literacy. The effectiveness of AI-driven software for lifestyle monitoring and personalized recommendations also depends heavily on patient motivation and adherence, which remain highly variable without direct clinical supervision. Overall, AI has considerable potential to transform the prevention and management of childhood obesity and its complications. However, current evidence remains insufficient to support widespread clinical implementation. Large, prospective, multi-center studies involving diverse pediatric populations, together with rigorous validation, health economic evaluations, and implementation research, are needed before AI-based prediction models can be safely and effectively integrated into routine pediatric clinical practice.

## Data Availability

No new data were created or analyzed in this study. Data sharing is not applicable to this article.

## Abbreviations

The following abbreviations are used in this manuscript:

T2DM	Type 2 Diabetes Mellitus
MASLD	Metabolic Dysfunction-Associated Steatotic Liver Disease
AI	Artificial Intelligence
ML	Machine Learning
SNPs	Single Nucleotide Polimorphisms
WHO	World Health Organization
BMI	Body Mass Index
COVID-19	Coronavirus Disease 2019
GWASs	Genome-Wide Association Studies
PYY	Peptide YY
GLP-1	Glucagon-Like Peptide-1
OXM	Oxintomodulin
CCK	Cholecystokinin
GIP	Gastric Inhibitory Polypeptide
PP	Pancreatic Polypeptide
OSAS	Obstructive Sleep Apnea Syndrome
BED	Binge Eating Disorder
MS	Metabolic Syndrome
MASH	Metabolic-Associated Steatohepatitis
DL	Deep Learning
NLP	Natural Language Processing
PCA	Principal Component Analysis
SVM	Support Vector Machine
ANN	Artificial Neural Network
CV	Computer Vision
CNNs	Convolutional Neural Networks
RNNs	Recurrent Neural Networks
EHRs	Electronic Health Records
NGS	Next-Generation Sequencing
CHICA	Child Health Improvement via Computer Automation
AAP	American Academy of Pediatrics
LLMs	Large Language Models
DTx	Digital Treatments
IOT	Internet of Things
NB	Naïve Bayes
DTs	Decision Trees
RF	Random Forest
KNN	K-Nearest Neighbor
LR	Logistic Regression
GB	Gradient Boosting
HDL	High-Density Lipoprotein
CART	Classification and Regression Tree
LASSO	Least Absolute Shrinkage and Selection Operator
XGBoost	eXtreme Gradient Boosting
